# Modeling extracellular matrix through histo-molecular gradient in NSCLC for clinical decisions

**DOI:** 10.3389/fonc.2022.1042766

**Published:** 2022-11-14

**Authors:** Camila Machado Baldavira, Tabatha Gutierrez Prieto, Juliana Machado-Rugolo, Jurandir Tomaz de Miranda, Lizandre Keren Ramos da Silveira, Ana Paula Pereira Velosa, Walcy Rosolia Teodoro, Alexandre Ab’Saber, Teresa Takagaki, Vera Luiza Capelozzi

**Affiliations:** ^1^ Department of Pathology, Faculty of Medicine, University of São Paulo, São Paulo, Brazil; ^2^ Health Technology Assessment Center, Clinical Hospital, Medical School of São Paulo State University, Botucatu, São Paulo, Brazil; ^3^ Rheumatology Division of the Hospital das Clínicas da Faculdade de Medicina da Universidade de São Paulo, Faculty of Medicine, University of São Paulo, São Paulo, SP, Brazil; ^4^ Division of Pneumology, Instituto do Coração (Incor), University of São Paulo Medical School (USP), São Paulo, Brazil

**Keywords:** lung cancer, extracellular matrix, epithelial-to-mesenchymal transition, WNT signaling pathway, glycosaminoglycans

## Abstract

Lung cancer still represents a global health problem, being the main type of tumor responsible for cancer deaths. In this context, the tumor microenvironment, and the extracellular matrix (ECM) pose as extremely relevant. Thus, this study aimed to explore the prognostic value of epithelial-to-mesenchymal transition (EMT), Wnt signaling, and ECM proteins expression in patients with non–small-cell lung carcinoma (NSCLC) with clinical stages I-IIIA. For that, we used 120 tissue sections from patients and evaluated the immunohistochemical, immunofluorescence, and transmission electron microscopy (TEM) to each of these markers. We also used *in silico* analysis to validate our data. We found a strong expression of E-cadherin and β-catenin, which reflects the differential ECM invasion process. Therefore, we also noticed a strong expression of chondroitin sulfate (CS) and collagens III and V. This suggests that, after EMT, the basal membrane (BM) enhanced the motility of invasive cells. EMT proteins were directly associated with WNT5A, and collagens III and V, which suggests that the WNT pathway drives them. On the other hand, heparan sulfate (HS) was associated with WNT3A and SPARC, while WNT1 was associated with CS. Interestingly, the association between WNT1 and Col IV suggested negative feedback of WNT1 along the BM. In our cohort, WNT3A, WNT5A, heparan sulfate and SPARC played an important role in the Cox regression model, influencing the overall survival (OS) of patients, be it directly or indirectly, with the SPARC expression stratifying the OS into two groups: 97 months for high expression; and 65 for low expression. In conclusion, the present study identified a set of proteins that may play a significant role in predicting the prognosis of NSCLC patients with clinical stages I-IIIA.

## 1 Introduction

Non-small-cell lung cancer (NSCLC) represents the most frequent malignant epithelial tumor of the lung. It accounts for 85% of the cases and mostly includes three histological subtypes: lung adenocarcinoma (ADC); lung squamous cell carcinoma (SqCC); and large cell carcinoma (LCC) (1). Globally, NSCLC remains the major cause of cancer mortality (2). In Brazil, the five-year overall survival (OS) is estimated at 18% (3). Radically resected NSCLC has a significant risk of progressing to distant metastasis, an outcome seen in 40% of the patients (4). One of the major issues associated with this process of tumor recurrence may be linked to occult metastases, which are still difficult to detect, even with current clinical advances (5).

Metastatic processes require malignant cells to invade the surrounding tissue *via* multiple steps. Two of these steps are of particular significance: the epithelial-mesenchymal transition process (EMT); and cell migration through the tissue to the vessels (6). To successfully complete both steps, tumor cells must express certain features, especially those that impact their ability to adhere to different molecules present in the extracellular matrix (ECM) and to other cell surfaces (7). Furthermore, since the ECM is remodeled, it is also relevant because it enables cell mobility, which results in metastasis (7).

The EMT process is relevant in NSCLC, which usually develops from the epithelial cells lining the bronchiolar and alveolar epithelium (8, 9). This is because it is through the EMT process that the cells change their epithelial phenotype in favor of a mobile mesenchymal phenotype (10). For this, epithelial cells interact with the basement membrane and undergo multiple biochemical changes that allow the alteration of cell phenotype. As a result, tumor cells acquire enhanced migratory capabilities, invasiveness, and significantly increased production of ECM molecules (11). Thus, ECM becomes crucial for cells to progress in this mesenchymal phenotype, thus reverting to their epithelial origins after secondary site invasion (12). Soon, tumor cells become able to re-epithelialize at the metastatic site. This is vital for colonization and development of metastatic extensions (13).

The ECM provides the cells with histoarchitectural support and anchoring. The ECM is composed of a complex network of highly cross-linked components, including fibrous proteins, glycoproteins, proteoglycans, and polysaccharides (14). The biomechanical and biochemical properties of the ECM regulate cell survival, proliferation, differentiation, and motility through the action of proteins such as SPARC, chondroitin sulfate (CS), heparan sulfate (HS), and collagens (15–18). The molecular changes that occur in the ECM have been potentially associated with invasive carcinoma. Furthermore, the modifications undergone by the ECM can modulate important signaling pathways in tissue morphogenesis, such as the Wnt signaling pathway. Abnormal signaling of this pathway is already associated with several types of cancer, where it exerts a tumorigenic effect (19, 20). In addition, this pathway has also been described as influencing the EMT process, which consequently acts on tumor growth and progression (19).

In this regard, several studies have investigated the ECM components individually. However, to the best of our knowledge, there are no studies investigating the structural components of the ECM combined with EMT behavior and the Wnt signaling pathway in NSCLC tissues. Thus, the present study analyzes the ECM patterns in different types of NSCLC and associates them with the expression of EMT markers and WNT proteins, and with the clinicopathologic features and outcome of patients.

## 2 Methods

### 2.1 Study cohort

We conducted a retrospective, longitudinal, and unicentric study on a consecutive series of patients with NSCLC who underwent surgery between 2004 and 2012 at the Thoracic Surgery Unit of Hospital das Clínicas, Instituto do Coração (InCor), and Instituto de Câncer de São Paulo (ICESP) linked to the University of São Paulo Medical School. We included chemo-naive patients with a histological diagnosis of NSCLC stage I, II or IIIA, and adequate tissue samples obtained from thoracic surgery. We excluded from the study patients treated with neoadjuvant chemotherapy and/or radiotherapy, palliative surgical procedure and the specimens inadequately fixed in paraffin.

We collected and managed patient data using REDcap electronic data capture tools at ICESP and included: sex, age, smoking history, histology, and disease stage – according to the 8th edition of the International Union for Cancer Control (UICC) TNM Classification of Malignant Tumors (21) –, as well as subsequent systemic or locoregional treatments, eventual recurrence, and death. All patients were followed up through monthly consultations with the oncologist and submitted to brain, chest, and abdominal CT scans every six months for the first five years, and annually thereafter. Overall survival (OS) served as the primary endpoint and was defined as first contact to death from recurrent lung cancer.

We carried the study out in accordance with the rules of Good Clinical Practice and the principles of the Declaration of Helsinki. The Internal Ethics Committees of all participating institutions approved this study protocol under number 150.443/2019.

### 2.2 Tissue microarray

The tissue microarray (TMA) slides were constructed with 120 samples of primary tumor tissue collected consecutively using three 1.5 mm tissue cores from the central, intermediate, and peripheral portions of the most representative tumor areas. An experienced pathologist had previously selected these areas and marked on a hematoxylin-eosin-stained sample. We used normal liver and kidney tissues to control and guide the slides and classified the tumors histologically according to the 2015 World Health Organization guidelines on lung cancer classification (22).

### 2.3 Immunohistochemistry and immunofluorescence assays

To perform the immunohistochemistry (IHC) and immunofluorescence (IF) assays, first we tested the immunostains on both whole tissue and TMA sections to ensure uniformity. We added negative controls to verify that the staining is specific using isotype antibody controls. Then, the TMA sections (N=120) were stained with immunoperoxidase and antibodies against: E-cadherin (1:100; Boster Biological), β-catenin (1:100; Santa Cruz), heparan sulfate (1:500; Santa Cruz), chondroitin sulfate (1:100, Santa Cruz), WNT1 (1:100, Santa Cruz), WNT3A (1:100, Abnova), WNT5A (1:400, Abnova), WNT5B (1:50, Santa Cruz), and SPARC (1:400, BIOSS).

To perform the immunofluorescence assay, we dewaxed the TMA sections (N=120) in xylol, hydrated in graded ethanol, and exposed them to a 0.3% hydrogen peroxide and formic acid solution to inhibit endogenous peroxidase activity. Antigen retrieval was accomplished using a citrate buffer solution at pH 9.0 and heated in a Pascal pressure cooker (125°C for 1 minute). Nonspecific sites were blocked with 5% bovine serum albumin (BSA) in phosphate buffer saline (PBS) for 30 minutes at room temperature. The specimens were incubated overnight at 4°C with antibodies against: E-cadherin (1:100; Boster Biological), β-catenin (1:100; Santa Cruz), heparan sulfate (1:500; Santa Cruz), chondroitin sulfate (1:100, Santa Cruz), anti-human collagen type I (1:700; Rockland Inc.), anti-human collagen type III (1:200; Rockland Inc.), anti-human collagen type IV (1:100; Dako), and anti-human collagen type V (1:1000; Rockland Inc.). These TMA sections were then washed in PBS with Tween 20 at 0.05% and incubated for 60 minutes at room temperature with Alexa 488-conjugated goat anti-mouse IgG (1:200, Invitrogen, Eugene, OR, USA) and Alexa 488-conjugated goat anti-rabbit IgG (1:200, Invitrogen, Eugene, OR, USA). For negative and autofluorescence controls, the sections were incubated with PBS and normal rabbit or mouse serum instead of the specific antibody. The nuclei were counterstained with 0.4 mM/mL 4’,6-Diamidino-2-Phenylindole, Dihydrochloride (DAPI; Molecular ProbesTM, Invitrogen, Eugene, OR, USA) for 15 minutes at room temperature. Finally, the specimens were mounted in buffered glycerol and their images were visualized in an immunofluorescence microscope (OLYMPUS BX51), and digitally scanned at ×20 magnification using a Pannoramic 250 whole slide scanner (3DHistech, Budapest, Hungary).

### 2.4 Quantification by image analysis

To measure the IHC expression of each different marker and quantify protein expression, the TMA slides were digitally scanned at ×40 magnification using a Pannoramic 250 whole slide scanner (3DHistech, Budapest, Hungary). The stained TMA sections were analyzed using QuPath (version 0.2.3; Centre for Cancer Research & Cell Biology, University of Edinburgh, Edinburgh, Scotland), an open-source image analysis software platform (23). During the scoring process, we assessed all cores to manually exclude any invalid samples (less than 10% of tumor per core or artifact).

QuPath allowed us to use a simple, automated, and semi-assisted method to quantify the TMAs. We first submitted each scanned TMA slide to a series of automated evaluations: staining vector analysis; total tissue area detection; tumor separation from non-tumor areas; and cellular detection. We then established the threshold of positivity for each of our markers through trial and error, and sent the cells considered to be positive to validation by an expert pathologist before applying them to the full array. Since GAGs are part of the extracellular matrix, – either on the cell surface or secreted in the form of PGs, as well as collagen types – the QuPath measurements we adopted were the percentage of positive tissue or expression within the tumor or stroma. Henceforth, we will refer to low expression whenever positive cell density is equal to or below the mean expression in the cohort, and to high expression whenever the positive cell density falls above this mean cut.

### 2.5 Transmission electron microscopy

Tissues were fixed in 2% glutaraldehyde buffer and post-fixed in 1% OsO4. The samples were then washed overnight in 0.9% saline solution containing uranyl and sucrose and soaked in Epon. Finally, the samples were stained with uranyl acetate and lead citrate and examined with a JEOL JEM-1010 electron microscope.

### 2.6 Data mining

The UALCAN platform (http://ualcan.path.uab.edu/), a user-friendly web resource, was used to analyze data from The Cancer Genome Atlas (TCGA) (24, 25) to investigate the relative expression of mRNA from our interest genes (E-cadherin, β-catenin, collagens type I, III, IV and V, WNT1, WNT3A, WNT5A, WNT5B, and SPARC) in ADC, SqCC, and normal samples. The mRNA expression level of the analyzed genes was normalized to transcription per million reads, and only a P-value not greater than 0.01, according to Student’s T-test, was significant. The UALCAN platform also was consulted to obtain the expression of ADC proteins present in the Clinical Proteomic Tumor Analysis Consortium (CPTAC) dataset (24, 26, 27). Protein expression was normalized according to Z-score. This database did not contain values for WNT1, WNT3A, and WNT5B proteins. Also, the database has not incorporated data for SqCC samples until this moment.

In this study, the clinicopathological characteristics of patients, as well as the expression levels of mRNA for the markers of interest were obtained in cBio Cancer Genomics (cBioPortal) (28, 29). This information was collected from the TCGA (Pan Cancer Atlas) database for both ADC and SqCC. To be consistent with our study model, we selected patients in pathological stages I, II, or IIIA. Data from this database that did not meet this criterion were excluded.

The prognostic significance value of proteins of interest in this study in NSCLC was evaluated using the Kaplan-Meier plotter database (30). Patient samples were divided into two cohorts, according to the median expression of each gene (high vs. low expression). The Kaplan-Meier plotter database calculated the log-rank P value and hazard ratio (HR) with 95% confidence intervals (CI).

The mRNA expression of enzymes involved in GAGs biosynthesis needs to be evaluated otherwise since their building blocks are polysaccharides synthesized in Golgi. Thus, the mRNA analysis for the GAGs on the databases described did not include them.

The String platform was consulted to reveal the functional interactions between the proteins evaluated in this present study and to map their protein-protein interaction (PPI) network (31, 32). We also used Metascape (33) to elucidate the function and biological processes involved in the enrichment of the genes corresponding to the proteins of our interest.

### 2.7 Data analysis

Since our data presented a distribution close to normal, we used the T-test, ANOVA, and Pearson’s chi-square test to associate protein expression, clinicopathologic characteristics, and histotypes. The Cox proportional hazards model was then used to analyze the association between OS rate and other covariances. Any parameters that were thought to be clinically relevant or had a P ≤ 0.02 in univariate analysis were considered for multivariate analysis. However, the data on the TCGA database presented a non-normal distribution, so we used non-parametric statistical tests instead. Finally, we used the statistical software IBM SPSS (version 22; Armonk, NY, USA) and RStudio to perform the analyses and plot the graphics. A P-value<0.05 was considered significant.

## 3 Results

### 3.1 Characterization of the NSCLC study cohort

**Table 1** shows the demographic and clinicopathologic characteristics of the 120 patients included in the study. Patients had a mean age of 65 years old (range, 30-80 years old) and were evenly distributed between male (66, 55%) and female (54, 45%). 71 patients (79.8%) had a history of tobacco use. We histologically classified most samples as ADC (73, 60.8%), followed by SqCC (40, 33.3%), and LCC (7, 5.8%). 91 patients (75.8%) were in T1 and T2 stage, with a greater proportion of patients (79, 65.8%) in the N0 lymph node stage. After surgical resection, the mean tumor size was 4.46 cm (range, 1 to 13 cm) and a pathological classification identified 39 patients in stage I (32.5%), 56 in stage II (46.7%), and 25 in stage IIIA (20.8%). 49 patients (42.2%) received adjuvant chemotherapy, and 32 patients (27.6%) received adjuvant radiotherapy. The mean follow-up was 57.3 months (range, 0-181) and, during this period, 31 patients (31.3%) were relapsed, and 65 patients died.

**Table 1:** Demographic and clinicopathologic characteristics of the patients (N=120).

Characteristics	Number (%) of Patients
**Age (years)**	
*Median (range)*	65 (30 – 88)
*≤65*	64 (53.3%)
*>65*	56 (46.7%)
**Gender**	
*Male*	66 (55.0%)
*Female*	54 (45.0%)
Smoke Status^a^	
*Smoker/Former-Smoker*	71 (79.8%)
*Non-Smoker*	18 (20.2%)
Smoking load^a^ (pack/years)	
*Mean (range)*	53.9 (1.5-150)
**Histological subtypes**	
*Adenocarcinoma*	73 (60.8%)
*Squamous cell carcinoma*	40 (33.3%)
*Large cell carcinoma*	7 (5.8%)
**T stage**†	
*T1*	30 (25.0%)
*T2*	61 (50.8%)
*T3*	23 (19.2%)
*T4*	6 (5.0%)
**N stage**†	
*N0*	79 (65.8%)
*N1* *N2*	24 (20.0%) 17 (14.2%)
Tumor size (cm)^a^	
*Mean (range)*	4.46 (1.0-13.0)
**Pathological Stage**†	
*I*	39 (32.5%)
*II*	56 (46.7%)
*IIIA*	25 (20.8%)
**Adjuvant treatment**	
*Chemotherapy* ^a^	
*No*	67 (57.8%)
*Yes*	49 (42.2%)
*Radiotherapy* ^a^	
*No*	84 (72.4%)
*Yes*	32 (27.6%)
Follow up (months)^a^	
*Mean (range)*	57.3 (0-181)
Relapse ^a^	31 (31.3%)
Status^a^	
*Death*	65 (65.7%)

a Some cases had missing follow-up information: smoke status (31); Smoking load (53); tumor size (2); Relapse (21); Chemotherapy (4); Radiotherapy (4); Status (21).

† According to 8th Edition International Association for the Study of Lung Cancer (21).

### 3.2 Epithelium-to-mesenchymal morphometric variables

As a first approach, we examined the epithelium-to-mesenchymal transition (EMT) phenotype through E-cadherin and β-catenin protein expression using IHC and IF. In addition, we observed the epithelium junctions and the ultrastructural pattern using TEM.

**Figure 1:**
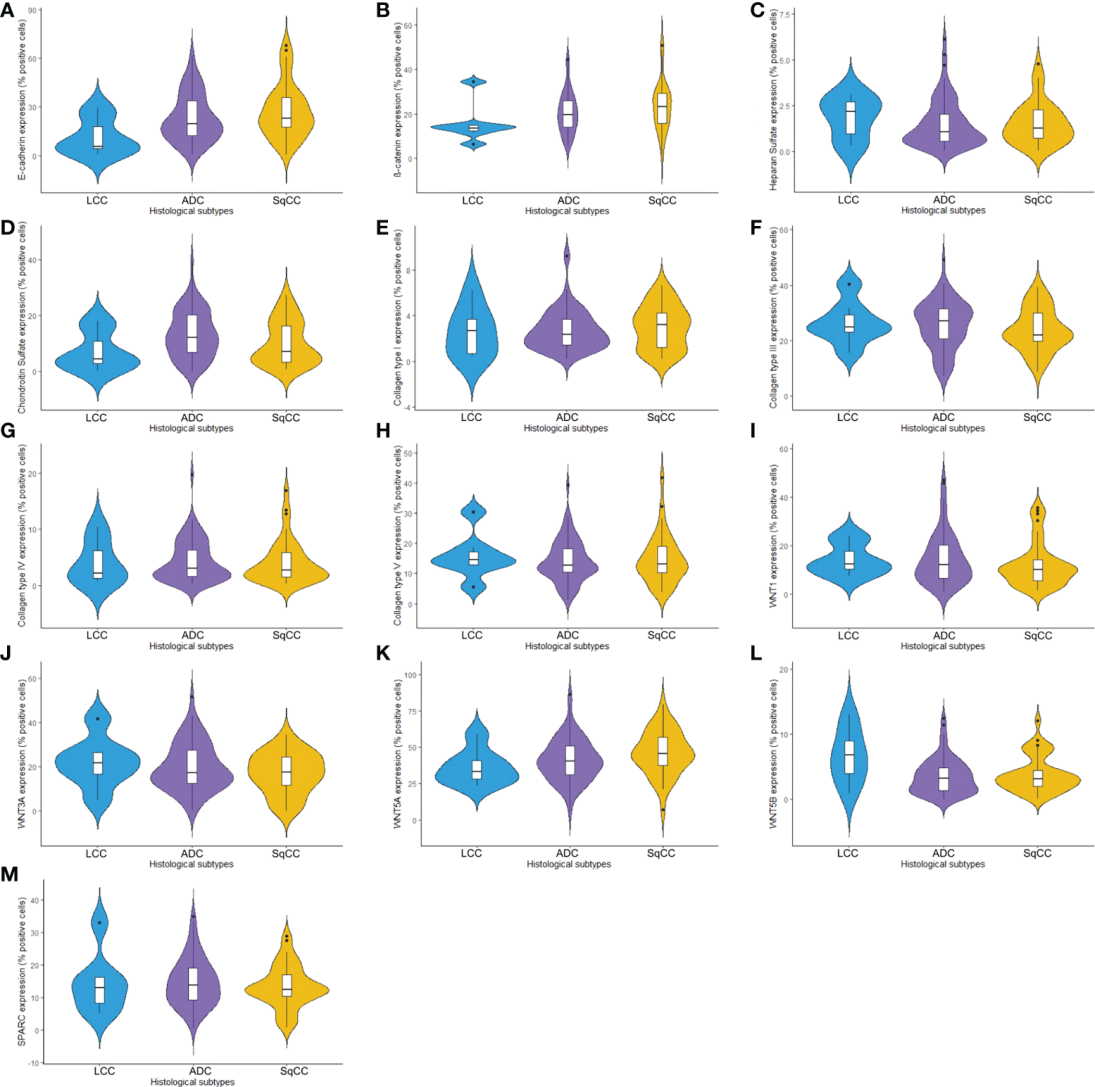
Violin plots graphs showing proteins of the epithelial-mesenchymal transition process, matricellular, and the Wnt signaling pathway expression analyzed by QuPath (N=120). The protein expression was demonstrated between three different histologic types in non-small cells lung cancer for **(A)** E-cadherin, **(B)** β-catenin, **(C)** Heparan sulfate, **(D)** Chondroitin sulfate, **(E)** Col I, **(F)** Col III, **(G)** Col IV, **(H)** Col V, **(I)** WNT1, **(J)** WNT3A, **(K)** WNT5A, **(L)** WNT5B, and **(M)** SPARC. LCC, large cell carcinoma; ADC, lung adenocarcinoma; SqCC, lung squamous cell carcinoma; Col, collagen type.

The mean expression of E-cadherin in tumor cells was 23.99% ± 1.49 positive cells. For β-catenin, the mean expression was 21.24% ± 0.90 positive cells. When we compared different histological subtypes, we observed that LCC samples expressed lower levels of both markers when compared to ADC and SqCC (**Figures 1A, B** **)**, with a significant difference between LCC and SqCC for E-cadherin expression (P=0.03); whereas ADC and SqCC had similar behaviors of expression for both markers. **Supplementary Table 1** shows the distribution of these two markers by histological subtypes.

**Figure 2:**
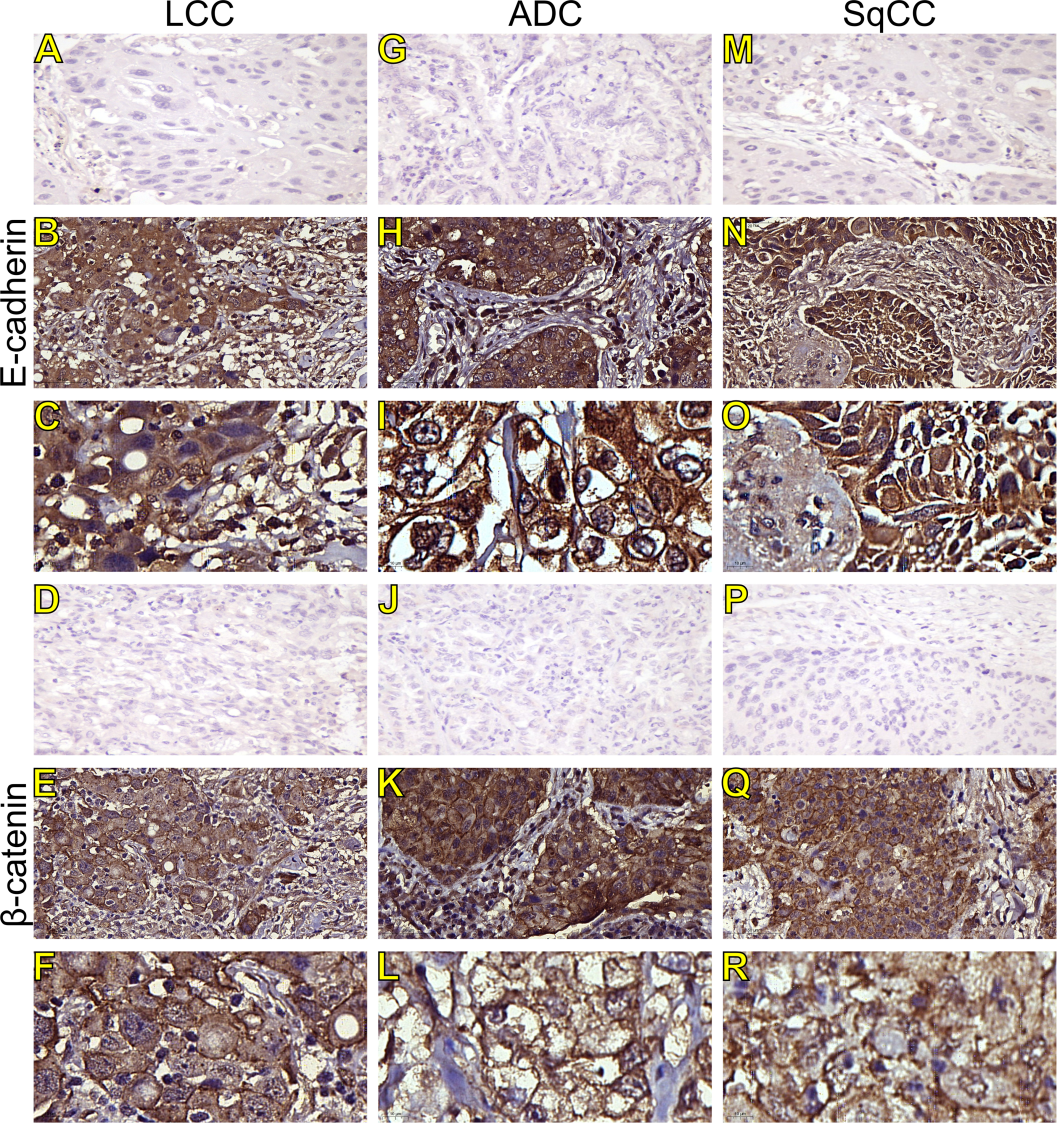
Immunohistochemistry expression of epithelial-to-mesenchymal transition-related markers in tumors and invasive groups of large cell carcinoma (**B, C**, E-cadherin; **E, F**, β-catenin; respectively), adenocarcinoma (**H, I**, E-cadherin; **K, L**, β-catenin; respectively) and squamous cell carcinoma (**N, O**, E-cadherin; **Q, R**, β-catenin; respectively) (N=120). For both markers, the first line illustrates the negative control **(A, D, G, J, M, P)**. Original magnification: 40X and 100X. LCC, large cell carcinoma; ADC, lung adenocarcinoma; SqCC, lung squamous cell carcinoma.

**Figure 3:**
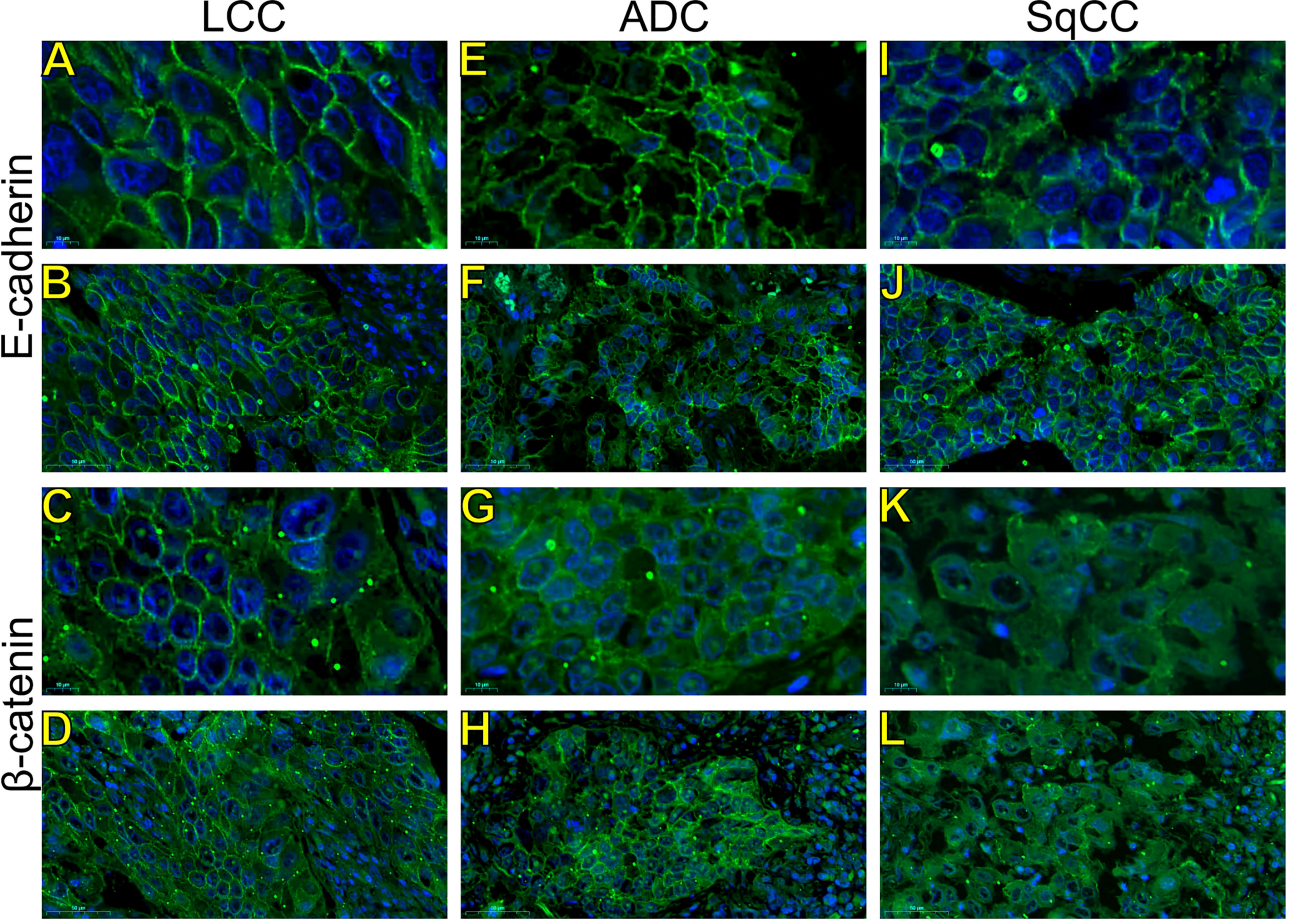
Expression of epithelial-to-mesenchymal transition-related markers. Images visualized under immunofluorescence microscope showing tumors and invasive groups of large cell carcinoma (**A, B**, E-cadherin; **C, D**, β-catenin; respectively), adenocarcinoma (**E, F**, E-cadherin; **G, H**, β-catenin; respectively) and squamous cell carcinoma (**I, J**, E-cadherin; **K, L**, β-catenin; respectively) (N=120). Original magnification: 100X and 40X. LCC, large cell carcinoma; ADC, lung adenocarcinoma; SqCC: lung squamous cell carcinoma.

**Figure 4:**
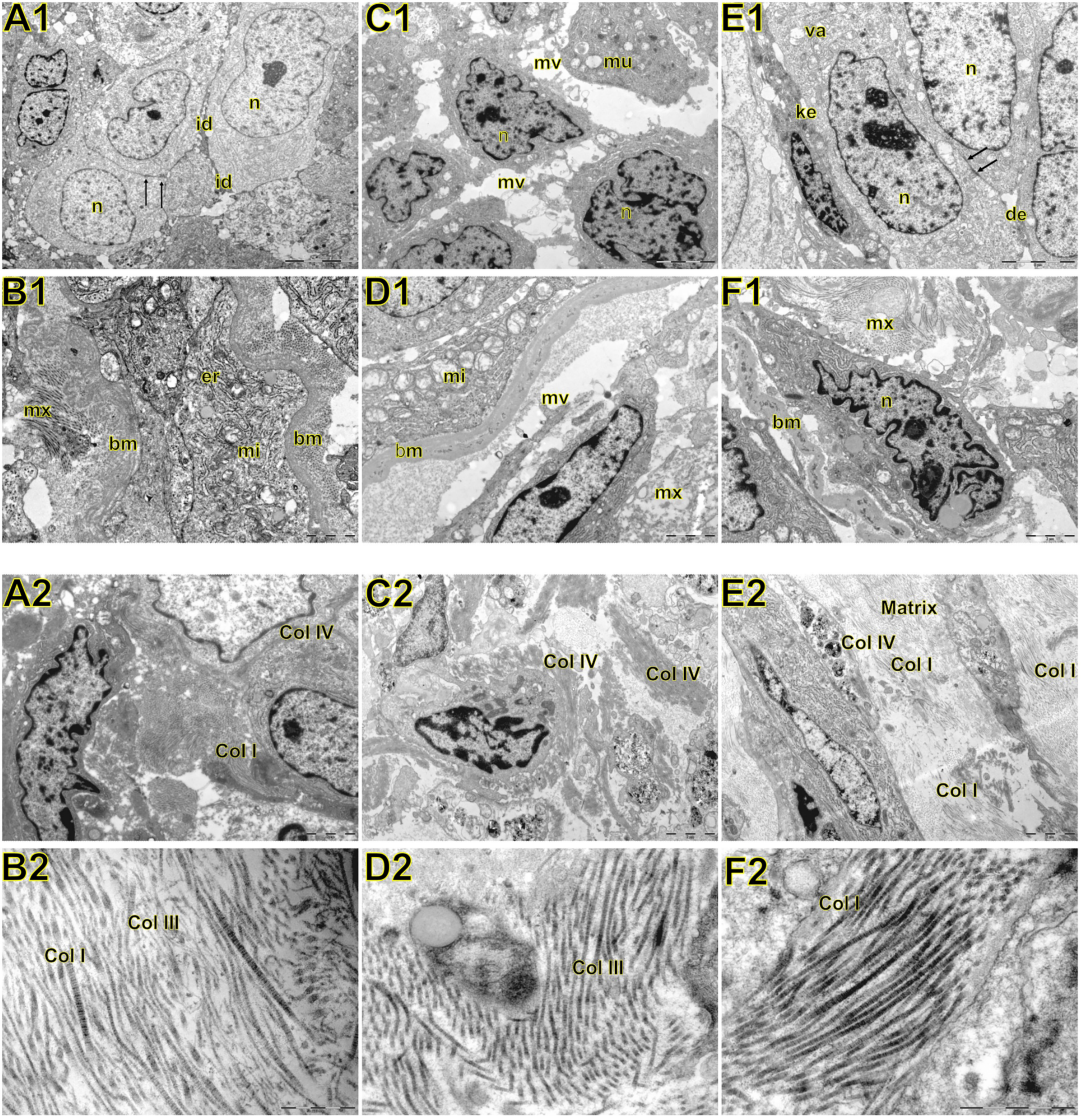
Transmission electron microscopy of large cell carcinoma **(A, B)**, adenocarcinoma **(C, D)**, and squamous cell carcinoma **(E, F)** visualized inside the tumor, basement membrane, and interstitial matrix. Above **A1:** large cell carcinoma showing large tumour cells with abundant light or little dark cytoplasm. Nuclei (n) are euchromatic and frequently display more or less deep invaginations with one to two prominent nucleoli. The cell membrane presented very short microvilli (mi) densely packed. Adjacent cells were interdigitated (id) with neighboring cells. In **B1**, note invasive large cell carcinoma with prominent organelles including mitochondria (mi) and endoplasmic reticulum (er) protruding and dissecting the basement membrane (bm) to invade the surrounding matrix (mx). **C1:** adenocarcinoma showing round cells with marked irregularity of nucleus, light cytoplasm, and interdigitation by sparse and short microvilli (mv). Mucin vacuoles (mu) can be visualized disperses in the cytoplasm. In **D1**, invasive cells protrude the basement membrane (bm) showing numerous organelles such as mitochondria (mi), and short microvilli projected toward the matrix (mx) assuming the fusiform phenotype. **E1**: squamous cell showing cells partially cohesive by junctional complexes (desmosomes, des). Some cells exhibit a pale and regular nucleus (n) with evident nucleolus. Adjoined cells are connected by numerous adherens junctions (arrows) in their lateral domains showing cellular polarity. Some autophagosomes (va) can be observed in the cytoplasm. The dark cells exhibit keratin filaments in the cytoplasm (ke). In **F1**, invasive squamous cells assume elongated form, marked irregularity of nucleus dissecting basement membrane (bm) and collagen fibers of the matrix (mx). Below **A2:** at low magnification large cell carcinoma showing abnormal large tumour cells with abundant light or little dark cytoplasm, densely cohesive. In **B2**, at high magnification note the interstitial matrix composed buy fibrillar collagens (fc) intermixed in a scant amorphous matrix. **C2**: a low magnification of adenocarcinoma showing aberrant round cells with marked irregularity of nucleus, thick basement membrane matrix in the junction of tumor cells with the interstitial matrix suggesting invadopodium. In **D2**, a high magnification shows fibrillar collagen (fc). **E2**: a low magnification showing elongated invasive squamous cells with the amorphous collagen (ac) along the basement membrane matrix. The interstitial matrix is abundant and composed of fibrillar collagen (fc) immersed in an amorphous matrix composed of heparan sulfate and chondroitin sulfate. In **F2**, a high magnification exhibited mainly thick and distorted fibers and microfibrils of fibrillar collagen (fc) in the interstitial matrix.

Morphologically, E-cadherin and β-catenin were expressed at the cell boundary in all three major histotypes (**Figures 2A–R** **;** **Figures 3A–L** **-** the negative control can be checked in **Supplementary Figure 1**), reflecting the ultrastructural pattern that is characterized by the presence of functional adherent junctions (**Figures 4A1–F1**).

In LCC, both EMT markers were arranged just beneath the plasma membrane, forming a thin cortical barrier around each malignant cell (**Figures 2C, F**, **3A, C**), in agreement with the short microvilli and cell interdigitation seen under TEM (**Figures 4A1–F1**). Finger-like projections interdigitated adjacent cells, whereas desmosomes linked small groups of cells (**Figure 4A1**). Notably, invasive LCC was characterized by long protrusions of cells dissecting the basement membrane (BM) to invade the surrounding matrix (**Figure 4B1**). LCC stained for E-cadherin and β-catenin both in the plasma membrane and in the cytoplasm (**Figures 2B, E**; respectively). This suggests a modest tumor proliferation and isolated invasion of the surrounding matrix.

In ADC, both EMT markers were even more evident in the plasma membrane (**Figures 2I, L**, **3E, G**). However, we also found the EMT markers in the cytoplasm of some cells. This indicates focal invadopodia, which attach to the matrix, as seen under TEM (**Figure 4C1**). Invasive adenocarcinomatous cells showed strong staining of E-cadherin and β-catenin in the cytoplasm and plasma membrane (**Figures 2H, K**), which indicates invasion of the surrounding stroma by groups of malignant cells with fusiform shape (**Figure 4D1**) and high tumor proliferation status.

Lastly, in SqCC, both EMT markers were highly evident and formed dots along the plasma membrane (**Figures 2O, R**), with invasive squamous cells also showing expression of E-cadherin and β-catenin in the cytoplasm (**Figures 2N, Q**). The TEM of SqCC samples showed adjacent cells connected by junctional complexes consisting of desmosomes (**Figure 4F1**). During the invasion of the surrounding matrix, we observed an evident spindle cell transformation with spindle-like projections extending into the BM, and the detachment of squamous cells.

The above results then suggest that, during EMT, the strong expression of E-cadherin and β-catenin seen at light microscopy reflects the ultrastructure of fragmentation and loss of continuity of adherent epithelial junctions. This enables ECM invasion by individual malignant cells, in the case of LCC, and cells groups, in the case of ADC and SqCC.

### 3.3 Extracellular matrix morphometric variables

Next, we examined morphometric variables linked to the ECM, including the glycosaminoglycans (GAGs) heparan sulphate (HS) and chondroitin sulphate (CS), and collagen type I (Col I), type III (Col III), type IV (Col IV), and type V (Col V).

**Figure 5:**
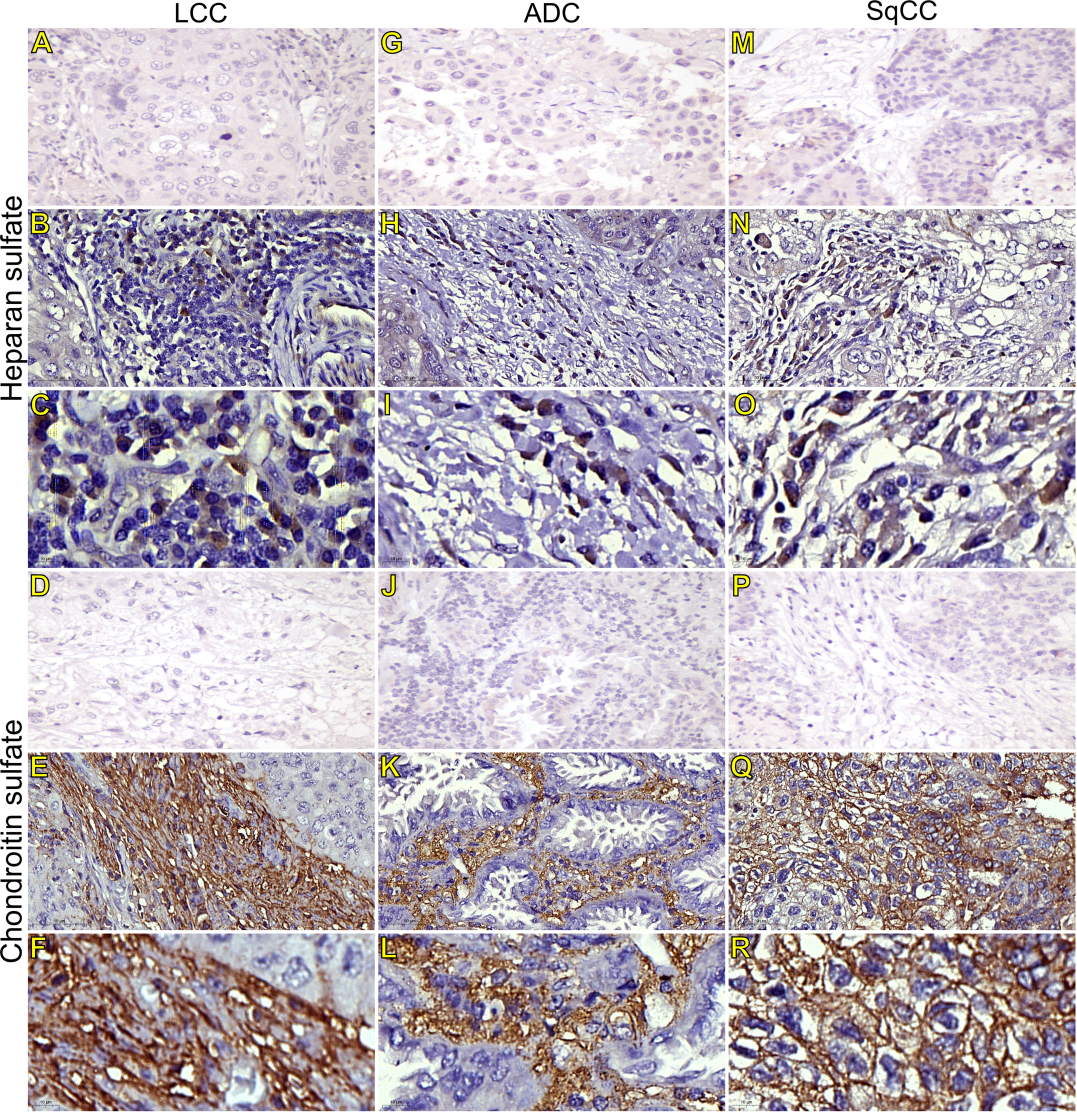
Immunohistochemistry expression of glycosaminoglycans markers in tumors and invasive groups of large cell carcinoma (**B**, **C**, heparan sulfate; **E**, **F**, chondroitin sulfate; respectively), in adenocarcinoma (**H**, **I**, heparan sulfate; **K**, **L**, chondroitin sulfate; respectively), and in squamous cell carcinoma (**N**, **O**, heparan sulfate; **Q**, **R**, chondroitin sulfate; respectively) (N=120). For both markers, the first line illustrates the negative control **(A, D, G, J, M, P)**. Original magnification: 40X and 100X. LCC, large cell carcinoma; ADC, lung adenocarcinoma; SqCC: lung squamous cell carcinoma.

Starting our analysis with the GAGs, we observed that the mean expression of HS was 1.55% ± 0.11 positive cells. This behavior coincided with weak immunostaining on all three major histotypes (**Figure 5**). Most ADC and SqCC samples showed lower expression of HS when compared with LCC, but with no statistical significance (**Figure 1C**; **Supplementary Table 1**).

In contrast, the mean expression of CS was 11.91% ± 0.75 positive cells, which agrees with the strong immunostaining similarly observed in all three histological subtypes (**Figure 1D**; **Supplementary Table 1**).

CS arrangement in LCC took place just beneath the plasma membrane. This CS arrangement formed a thick cortical barrier around it (**Figures 5E, F**), in agreement with the thick BM seen under TEM (**Figure 4A2**). Furthermore, there was strong CS staining in both the plasma membrane and in the cytoplasm (**Figures 5E, F**; respectively), a sign of rapid proliferation of the malignant cells invading the interstitial ECM.

In ADC, the presence of CS was even more evident in the BM (**Figures 5K, L**), although also being found in the cytoplasm of some cells. This indicates focal invadopodia, which attach to the BM, as described under TEM (**Figure 4C2**). Invasive adenocarcinomatous cells presented intense CS staining in the cytoplasm and plasma membrane (**Figures 5K, L**) The intense CS staining shows a rapid invasion of the surrounding ECM by cell groups with high tumor proliferation status. In contrast, CS in invasive SqCC was considerably more evident than in others histotypes, forming a thick barrier along the BM with strong CS expression in the cytoplasm (**Figures 5Q, R**).

When we quantify the different collagen types in this context, the mean collagen expression was 2.80% ± 0.16 positive fibers for Col I, 25.04% ± 0.76 positive fibers for Col III, 4.29% ± 0.33 positive fibers for Col IV, and 14.41% ± 0.66 positive fibers for Col V. The expression of all four makers was similar across the three histological subtypes (**Figures 1E–H**, **Supplementary Table 1**).

**Figure 6:**
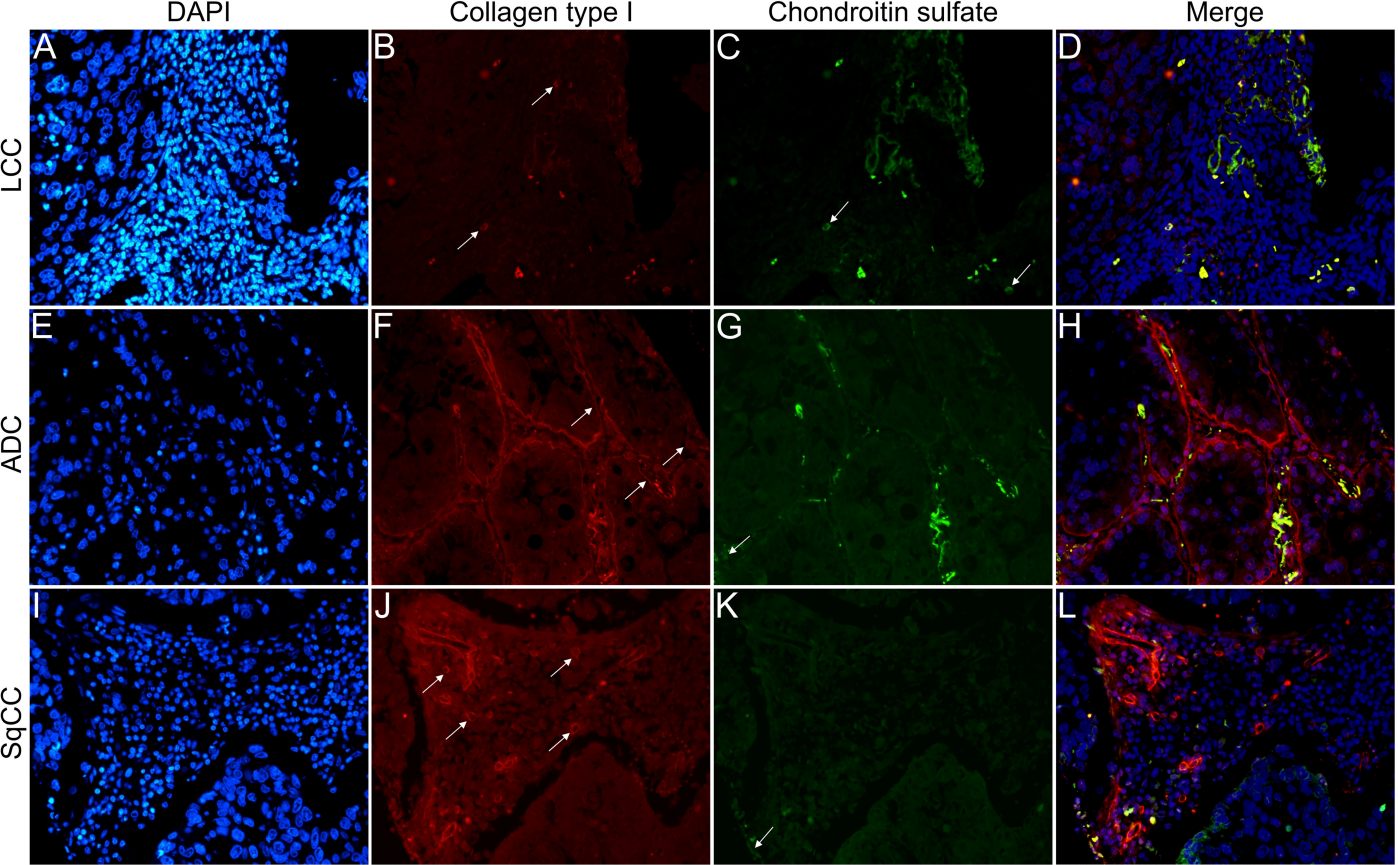
Co-analysis of immunofluorescence of chondroitin sulfate (green; **C**, **G**, **K**) and collagen type I (red; **B**, **F**, **J**) in three different histological subtypes of non-small cell lung carcinoma (N=120). The stained nuclei are represented in blue (DAPI; **A**, **E**, **I**). Images **D**, **H**, **L** represent the merge of the same field of these three stains. White arrows indicate positive expression of the markers. Original magnification: 40x. LCC, large cell carcinoma; ADC, lung adenocarcinoma; SqCC: lung squamous cell carcinoma.

**Figure 7:**
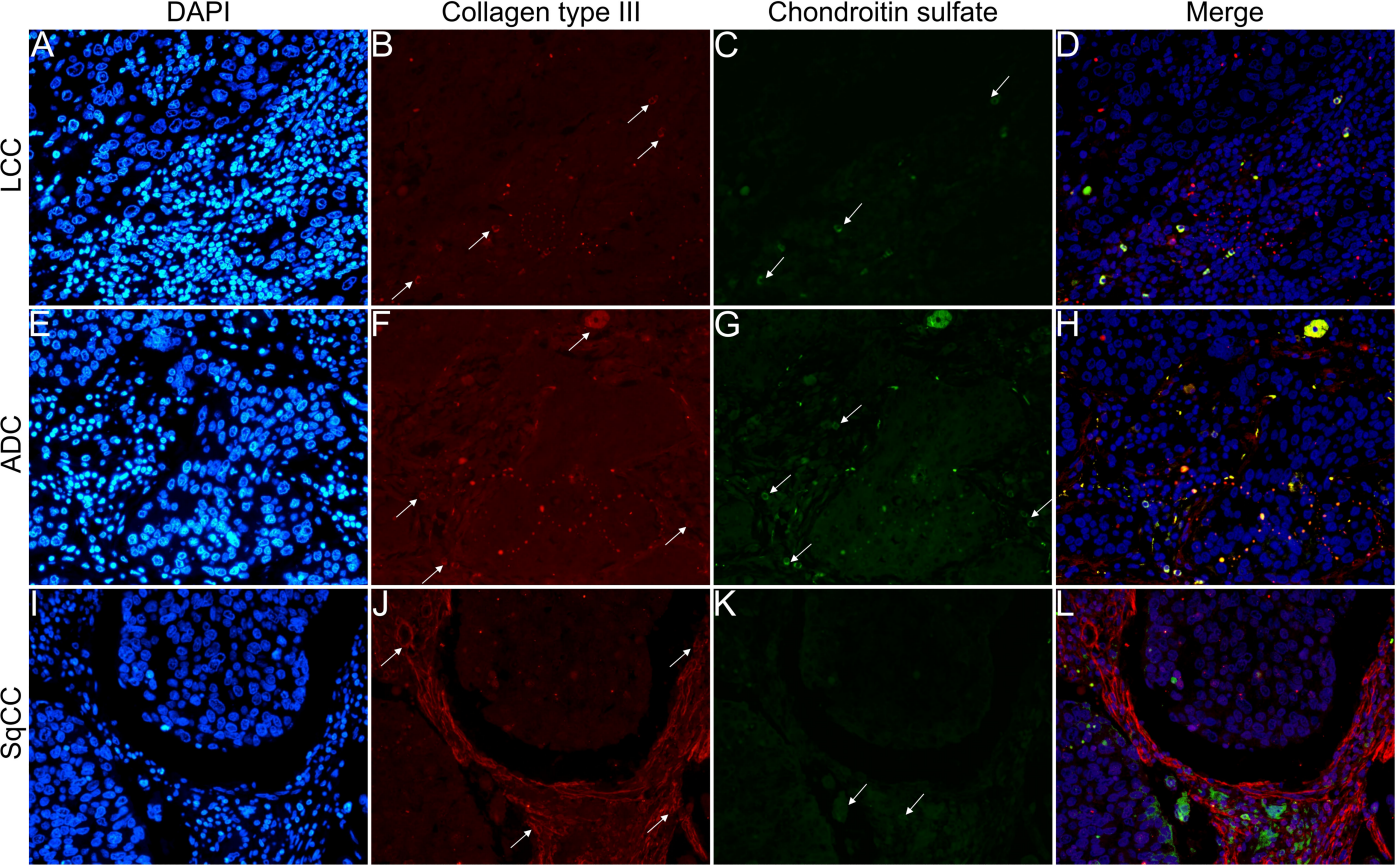
Co-analysis of immunofluorescence of chondroitin sulfate (green; **C**, **G**, **K**) and collagen type III (red; **B**, **F**, **J**) in three different histological subtypes of non-small cell lung carcinoma (N=120). The stained nuclei are represented in blue (DAPI; **A**, **E**, **I**). Images **D**, **H**, **L** represent the merge of the same field of these three stains. White arrows indicate positive expression of the markers. Original magnification: 40x. LCC, large cell carcinoma; ADC, lung adenocarcinoma; SqCC: lung squamous cell carcinoma.

**Figure 8:**
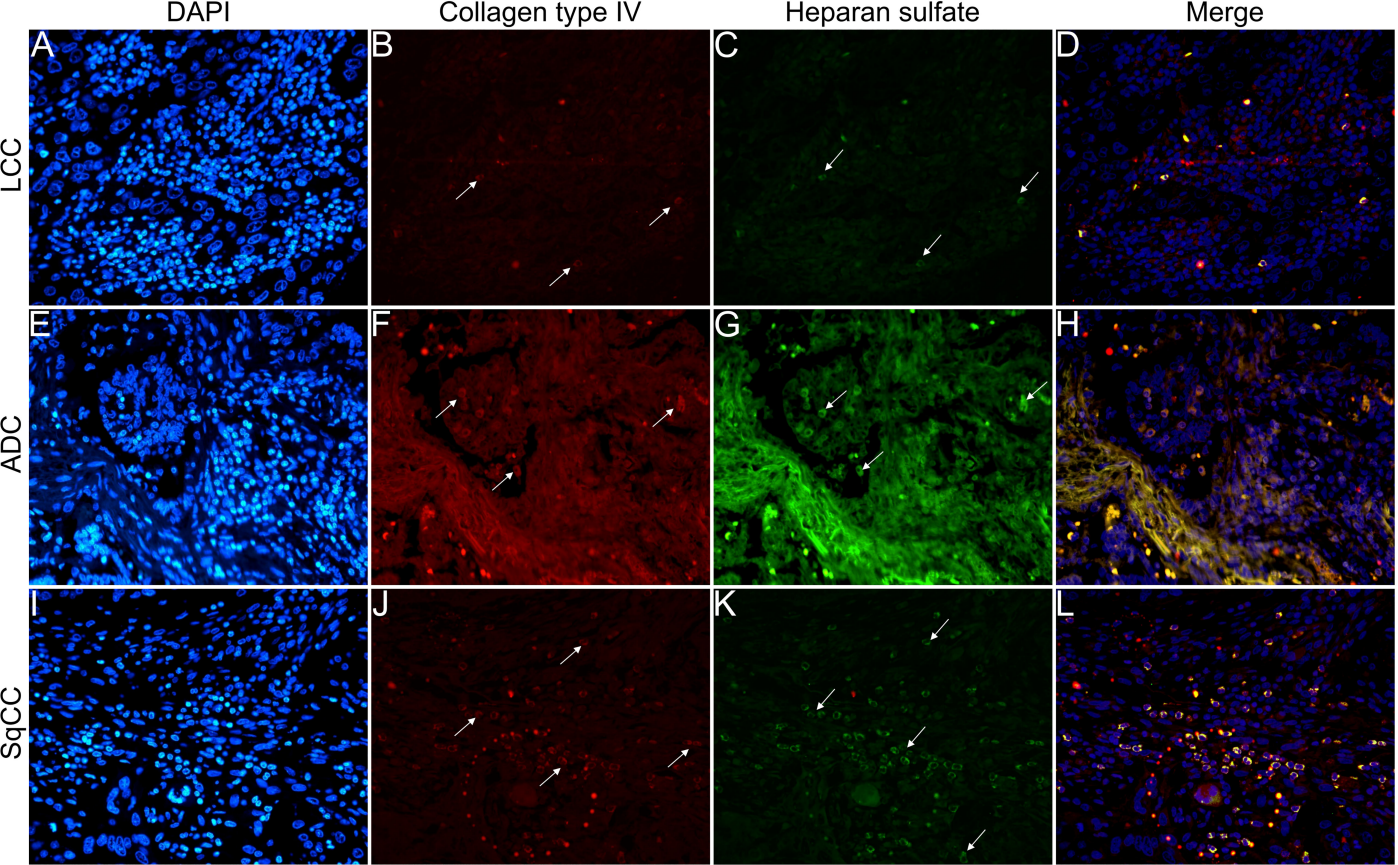
Co-analysis of immunofluorescence of heparan sulfate (green; **C**, **G**, **K**) and collagen type IV (red; **B**, **F**, **J**) in three different histological subtypes of non-small cell lung carcinoma (N=120). The stained nuclei are represented in blue (DAPI; **A**, **E**, **I**). Images **D**, **H**, **L** represent the merge of the same field of these three stains. White arrows indicate positive expression of the markers. Original magnification: 40x. LCC, large cell carcinoma; ADC, lung adenocarcinoma; SqCC: lung squamous cell carcinoma.

**Figure 9:**
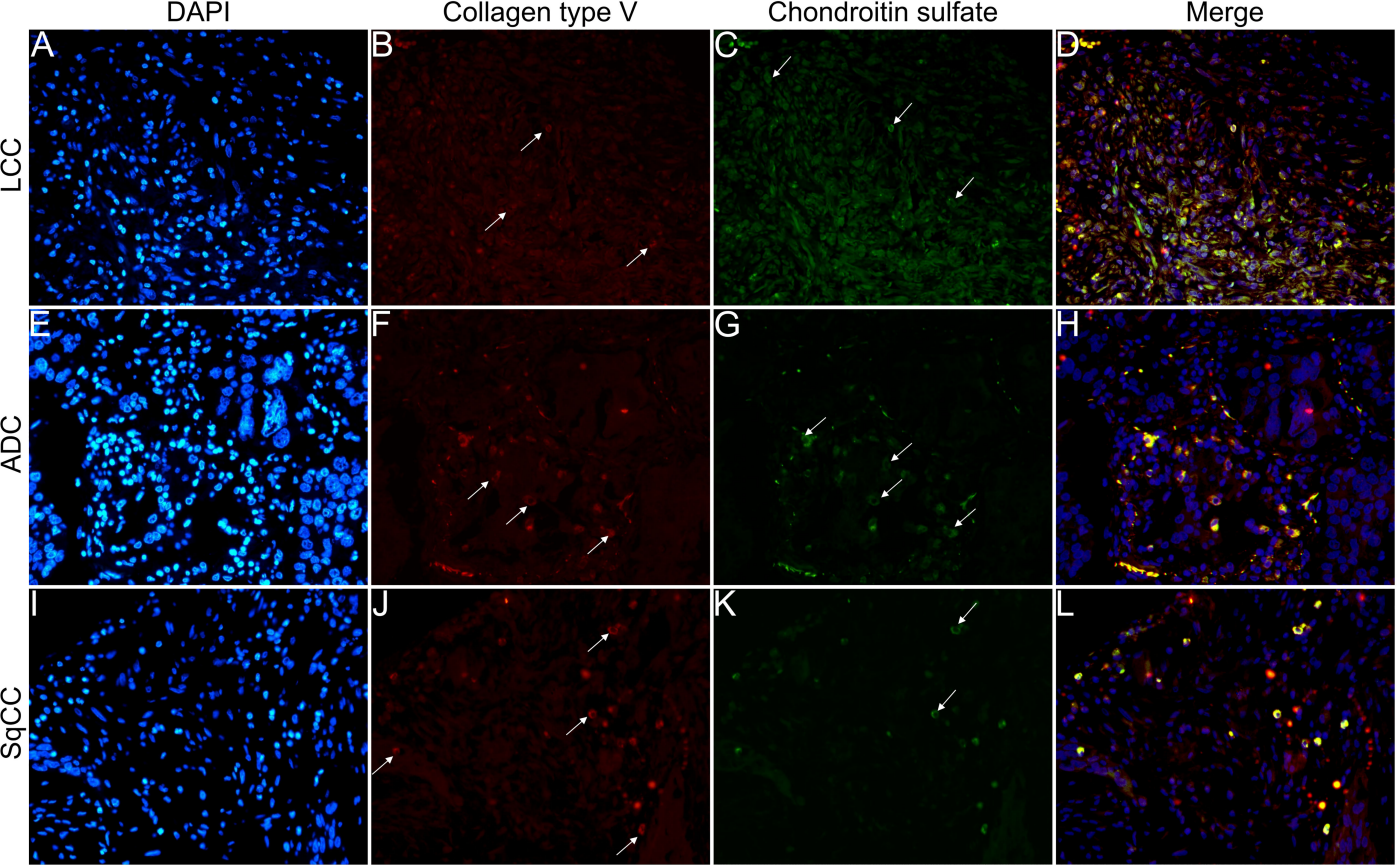
Co-analysis of immunofluorescence of chondroitin sulfate (green; **C**, **G**, **K**) and collagen type V (red; **B**, **F**, **J**) in three different histological subtypes of non-small cell lung carcinoma (N=120). The stained nuclei are represented in blue (DAPI; **A**, **E**, **I**). Images **D**, **H**, **L** represent the merge of the same field of these three stains. White arrows indicate positive expression of the markers. Original magnification: 40x. LCC, large cell carcinoma; ADC, lung adenocarcinoma; SqCC: lung squamous cell carcinoma.

**Figures 6** **–** **9** show the IF co-analyses of CS/HS and collagen types (the respectively negative control can be found in **Supplementary Figure 2**). The interstitial matrix of ADC and SqCC showed a strong reddish fluorescence of Col I fibers; conversely, CS is represented by a strong greenish fluorescence along the BM in ADC and LCC (**Figure 6**). While Col III fibers are seen as in a strongly reddish fluorescent color in the interstitial matrix of LCC, ADC, and SqCC (**Figures 7B, F, J** **)**, CS showed a weak greenish fluorescence in the BM of the three histotypes (**Figures 7C, G, K** **)**. The intensity of refringence of Col IV (red) and HS (green) in the BM was strong in ADC and weak in SqCC and LCC (**Figure 8**). Notably, Col V (red) and CS (green) were more fluorescent in LCC compared to ADC and SqCC (**Figure 9**).

The above results suggest that, after EMT, the strong expression of CS, with the strong refringence of Col III and Col V, may enhance the motility of invasive cells from the three histotypes through the BM into the interstitial ECM.

### 3.4 Wnt signaling pathway and SPARC morphometric expression

We then evaluated Wnt signaling through WNT1, WNT3A, WNT5A, and WNT5B expression in NSCLC histotypes. The Wnt protein family triggers a relevant cascade which regulates development and is associated with cancer. We also examined the morphometric variables of SPARC, a multifunctional glycoprotein involved with the EMT, ECM remodeling, and Wnt proteins.

**Figure 10:**
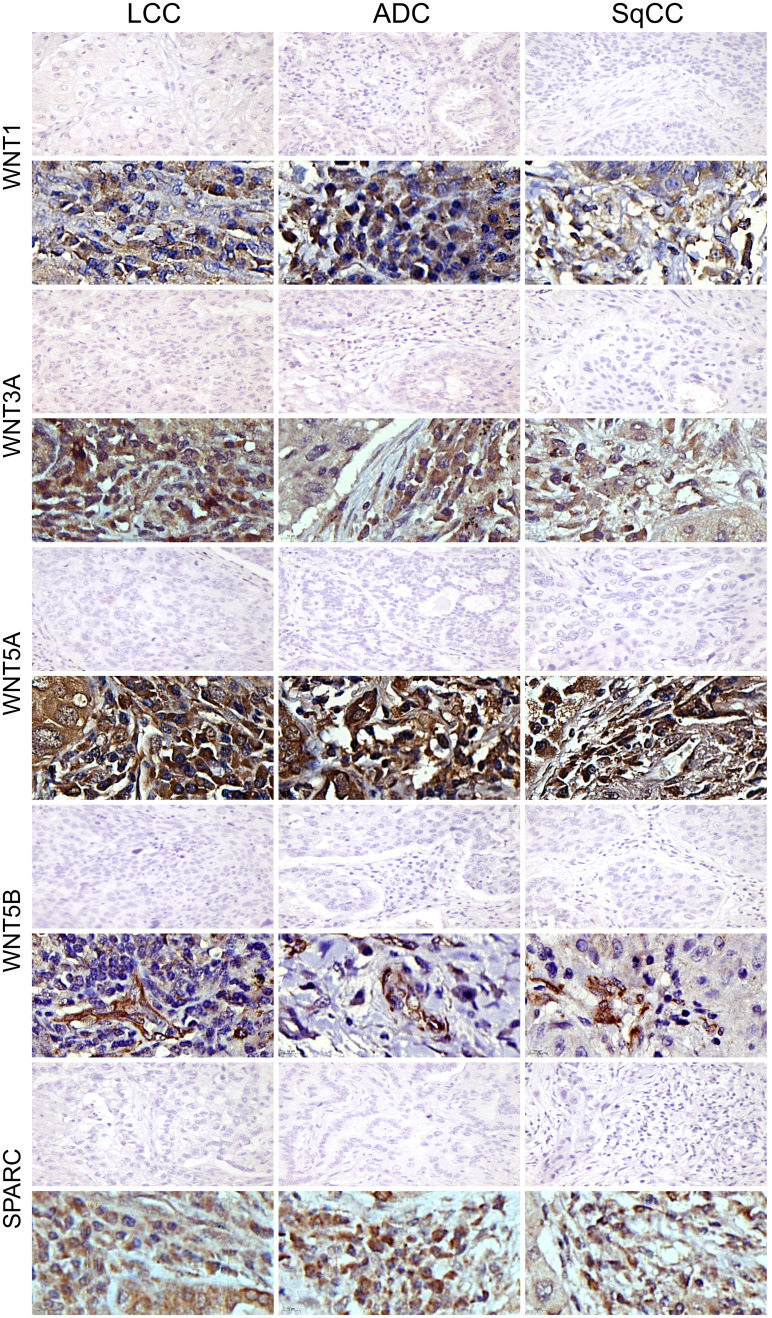
Immunohistochemistry expression of WNTs and SPARC in large cell carcinoma, adenocarcinoma, and squamous cell carcinoma (N=120). For both markers, the first line illustrates the negative control. Original magnification: 40X and 100X. LCC, large cell carcinoma; ADC, lung adenocarcinoma; SqCC: lung squamous cell carcinoma.

The mean expression of WNT1, WNT3A, WNT5A, and WNT5B by tumor cells was respectively 13.55% ± 0.89, 19.16% ± 0.94, 42.47% ± 1.35, and 3.74% ± 0.26 positive cells, which coincides with a moderate expression of WNT1 and WNT3A, a weak expression of WNT5B, but an intense cytoplasmic expression of WNT5A in the three histologic subtypes (**Figure 10**). However, only the expression of WNT5A showed a significant statistical difference between histological subtypes (SqCC versus non-squamous tumors, **Figure 1K**; **Supplementary Table 1**).

For SPARC, the mean expression in tumor stroma was 14.24% ± 0.65 positive cells, with similar proportions among histotypes. Although cytoplasmic staining was predominantly positive in the stroma cells in all NSCLC cases, we also observed weak SPARC staining in tumor cells across all three histological subtypes (**Figure 10**). There was no statistical difference in SPARC levels between the three histotypes (**Figure 1M**, **Supplementary Table 1**).

These findings show the importance of WNT5A expression across histotypes, and its possible association with tumor progression.

### 3.5 Correlation between EMT, WNTs, SPARC, and ECM morphometric variables

The next step was to explore whether the WNT proteins or SPARC mechanistically orchestrated the phenotypic and molecular changes in NSCLC. This would suggest EMT and ECM as intrinsic links between development and cancer progression.

**Figure 11:**
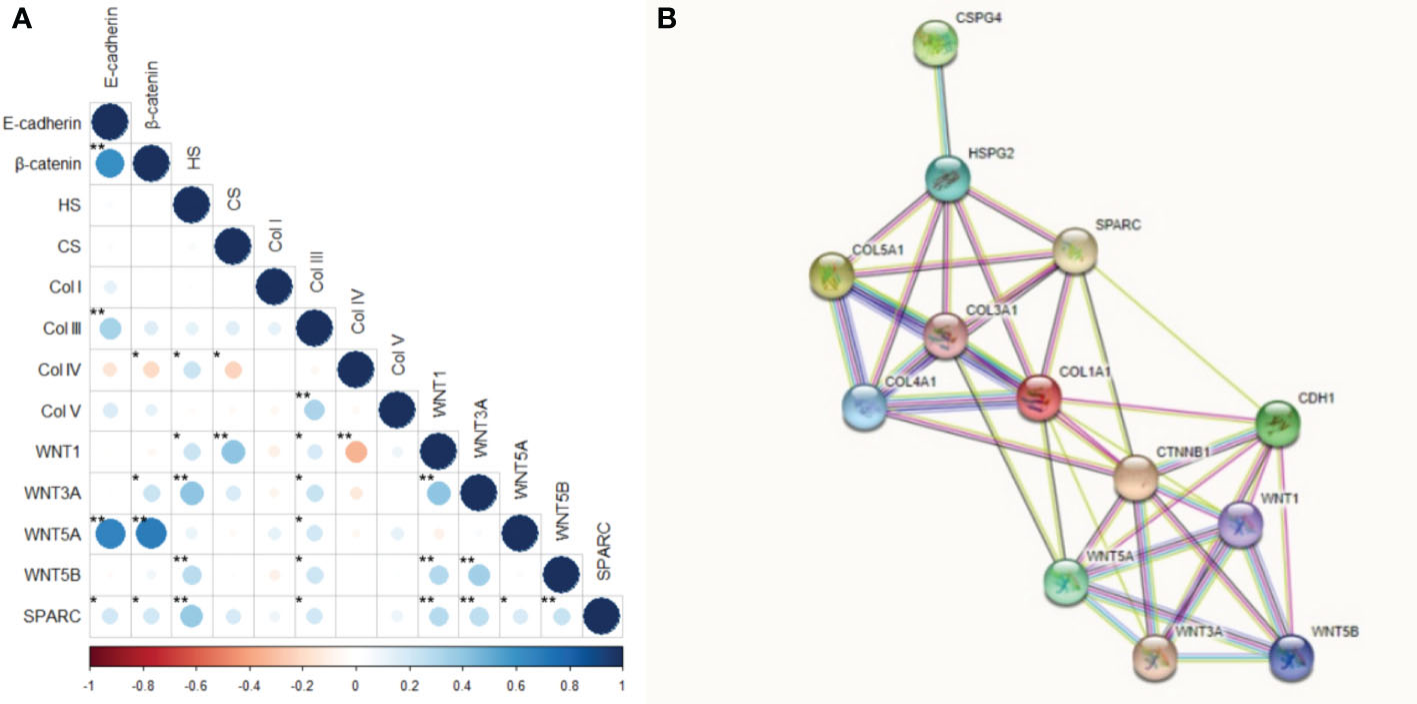
Correlation and protein-protein interaction network. **(A)** Correlation between the interest markers. The graduation of colors represents the positive or negative correlation. The size of the dot represents the Spearman’s rho, larger dots have values closer to |1|, therefore, more strong correlation. *P<0.05; **P<0.01. **(B)** Protein-protein interaction network obtained into the STRING tool for our interest proteins.

**Figure 11** shows the correlation between the tumor and stroma morphometric variables. E-cadherin correlated strongly with β-catenin (ρ=0.617, P<0.001) and WNT5A (ρ=0.672, P<0.001), and moderately with Col III (ρ=0.336, P<0.001). β-catenin also correlated strongly with WNT5A (ρ=0.693, P<0.001).

HS moderately correlated with WNT3A (ρ=0.401, P<0.001) and SPARC (ρ=0.376, P<0.001), while CS moderately correlated with WNT1 (ρ=0.406, P<0.001). Col IV was moderately inversely associated with WNT1 (ρ =-0.346, p<0.001), whereas Col V moderately correlated with Col III (ρ=0.329, P<0.001). WNT1 also had a moderate correlation with WNT3A (ρ=0.403, P<0.001), and WNT3A moderately correlated with WNT5B (ρ=0.340, P<0.001).

These results suggest the WNT5A pathway drove the EMT and the synthesis of both Col III signaling and Col V. On the other hand, WNT3A and SPARC regulated HS, while WNT1 directly regulated CS. A WNT1 negative feedback loop controlled the synthesis of Col IV along the BM.

### 3.6 Associations between clinicopathological features and morphometric variables

**Table 2** shows the clinicopathological characteristics stratified by E-cadherin, β-catenin, WNTs signaling proteins, and SPARC. Statistical significance was found between lower E-cadherin expression and younger patients (P=0.04), patients with LCC (P=0.03), and tumors smaller than 3 cm (P=0.02). We also found statistical significance between tumors smaller than 3 cm and a higher WNT1 expression (P=0.05) and lower WNT5A expression (P=0.04). Patients with LCC showed higher WNT5B expression (P=0.01).

**Table 2:** Association between clinicopathologic characteristics and mean expression (% positive expression) of E-cadherin, β-catenin, WNTs signaling proteins, and SPARC (*t* test and ANOVA, P<0.05).

Characteristics	E-cadherin	β-catenin	WNT1	WNT3A	WNT5A	WNT5B	SPARC
**Age (years)**							
≤65	21.35 ± 1.66*	21.01 ± 1.26	13.89 ± 1.29	17.54 ± 1.27&	41.56 ± 1.82	3.86 ± 0.39	14.05 ± 0.84
> 65	**27.00 ± 2.30**	21.51 ± 1.32	13.19 ± 1.26	20.96 ± 1.39	43.52 ± 2.03	3.61 ± 0.36	14.47 ± 1.03
**Gender**							
Male	25.24 ± 1.91	21.10 ± 1.14	12.11 ± 1.04&	18.57 ± 1.25	43.21 ± 1.84	3.79 ± 0.32	13.83 ± 0.86
Female	22.46 ± 2.08	21.43 ± 1.48	15.39 ± 1.53	19.92 ± 1.46	41.58 ± 2.01	3.69 ± 0.43	14.77 ± 1.01
**Tobacco History**							
Smoker/Former-smoker	28.15 ± 1.79	23.06 ± 1.23	13.28 ± 1.28	18.45 ± 1.18	44.61 ± 1.69	3.74 ± 0.33	14.62 ± 0.86
Non-smoker	27.85 ± 4.10	22.00 ± 2.22	13.80 ± 2.52	21.27 ± 2.64	43.16 ± 4.05	3.16 ± 0.63	13.44 ± 1.53
**Histotypes**							
ADC	**23.23 ± 1.70**	20.59 ± 1.08	14.24 ± 1.22	20.00 ± 1.26	41.04 ± 1.71	**3.45 ± 0.32**	14.69 ± 0.86
SqCC	**27.57 ± 2.63**	23.48 ± 1.73	12.19 ± 1.48	17.11 ± 1.49	46.18 ± 2.39	**3.79 ± 0.44**	13.42 ± 1.03
LCC	11.52 ± 4.17*#	15.54 ± 3.35	14.17 ± 2.45	22.13 ± 4.30	36.28 ± 4.58	6.61 ± 1.57*##	14.35 ± 3.53
**T stage**							
T1	21.55 ± 2.55	24.53 ± 1.94	15.76 ± 2.32	20.12 ± 2.05	41.37 ± 2.67	3.93 ± 0.60	13.61 ± 1.18
T2	24.78 ± 2.06	19.78 ± 1.09	13.50 ± 1.12	20.24 ± 1.24	42.64 ± 1.87	3.83 ± 0.32	15.26 ± 1.01
T3	25.20 ± 3.58	20.86 ± 2.24	11.63 ± 1.89	16.51 ± 2.30	42.27 ± 3.45	3.32 ± 0.65	13.24 ± 1.16
T4	23.57 ± 3.92	21.55 ± 6.23	10.81 ± 2.92	14.08 ± 3.86	47.14 ± 5.70	3.63 ± 1.94	11.08 ± 3.14
**N stage**							
N0	25.22 ± 1.79	22.11 ± 1.13	13.55 ± 1.21	19.55 ± 1.13	42.37 ± 1.63	3.69 ± 0.31	14.81 ± 0.77
N1	20.11 ± 2.85	19.66 ± 2.29	13.63 ± 1.59	20.31 ± 2.49	41.77 ± 3.74	4.44 ± 0.73	12.59 ± 1.63
N2	23.73 ± 3.65	19.42 ± 0.91	13.48 ± 1.98	15.90 ± 2.24	43.96 ± 2.69	3.03 ± 0.59	13.59 ± 1.81
**Pathologic stage**							
I	26.39 ± 2.66	24.03 ± 1.65	16.48 ± 2.00	20.71 ± 1.64	43.21 ± 2.46	3.85 ± 0.47	15.85 ± 1.20
II	23.02 ± 2.08	19.99 ± 1.30	11.83 ± 2.00&&	19.59 ± 1.34	41.78 ± 2.05	3.91 ± 0.33	13.95 ± 0.90
IIIA	22.05 ± 2.75	19.38 ± 1.89	12.19 ± 1.42	15.60 ± 2.29	42.97 ± 2.77	3.22 ± 0.74	12.36 ± 1.50
**Tumor size**							
≤3 cm	20.60 ± 1.86*	22.18 ± 1.41	15.80 ± 1.66*	21.29 ± 1.54	39.42 ± 2.08*	4.07 ± 0.42	15.15 ± 1.05
>3 cm	**27.02 ± 1.96**	20.68 ± 1.20	**12.02 ± 0.96**	17.84 ± 1.17&	**45.05 ± 1.76**	3.60 ± 0.34	13.75 ± 0.85
**Relapse**							
No	23.96 ± 1.90	21.70 ± 1.25	14.02 ± 1.28	19.19 ± 1.20	42.60 ± 1.90	3.90 ± 0.37	15.09 ± 0.88
Yes	24.38 ± 2.71	19.98 ± 1.94	12.79 ± 1.37	17.05 ± 1.87	42.85 ± 2.66	3.47 ± 0.44	12.98 ± 1.29
**Adjuvant treatment**							
*Chemotherapy*							
No	22.39 ± 1.84	21.40 ± 1.23	14.27 ± 1.33	19.75 ± 1.24	41.45 ± 1.87	3.89 ± 0.36	15.48 ± 0.92
Yes	26.60 ± 2.33	21.24 ± 1.46	12.85 ± 1.26	18.97 ± 1.56	43.61 ± 2.07	3.72 ± 0.41	12.97 ± 0.91$
*Radiotherapy*							
No	24.48 ± 1.94	22.06 ± 1.02	13.93 ± 1.16	19.78 ± 1.17	42.57 ± 1.59	3.86 ± 0.32	14.55 ± 0.76
Yes	23.33 ± 3.09	19.45 ± 2.06	12.95 ± 1.45	18.48 ± 1.75	41.80 ± 2.82	3.71 ± 0.48	14.08 ± 1.39

*P>0.05; #SqCC vs. LCC - P=0.03; ##ADC vs. LCC - P=0.01, and SqCC vs. LCC - P=0.04; &P=0.07; &&Pathological Stage I vs. II – P=0.06; $P=0.06

ADC, lung adenocarcinoma; SqCC: lung squamous cell carcinoma; LCC, large cell carcinoma.

Bolded values refer to a P-value with statistical significance (P<0.05).

We found borderline significance between lower WNT1 expression in male patients (P=0.07) and stage II tumor versus stage I (P=0.06). This borderline significance suggests that increased WNT1 expression occurs at earlier stages of carcinogenesis, which then decreases as the tumor grows. There was also borderline significance between lower expression of WNT3A and younger patients (P=0.07), and between tumors larger than 3 cm (P=0.07). Finally, lower SPARC expression had a borderline significance in patients who had received adjuvant chemotherapy (P=0.07).

We did not find a significant association between tobacco history, T stage, N stage, relapse, and the makers under investigation. β-catenin expression failed to show any significant association with clinicopathologic characteristics.

**Table 3** shows the association between clinicopathological features, GAGs, and collagen types. There was a significant association between lower CS expression and tumors larger than 3 cm (P=0.05). In turn, lower Col IV expression was associated with younger (P=0.05) and female (P=0.004) patients. Tumors in the N2 stage tended to express lower Col V in the stroma (p=0.05) when compared to N0, as did tumors from patients who received adjuvant chemotherapy (P=0.03).

**Table 3:** Association between clinicopathologic characteristics and mean expression (% positive expression) of GAGs and collagen types (*t* test and ANOVA, P<0.05).

Characteristics	HS	CS	Col I	Col III	Col IV	Col V
**Age (years)**						
≤65	1.52 ± 0.16	12.21 ± 1.06	2.85 ± 0.21	23.67 ± 1.00	3.64 ± 0.35*	13.63 ± 0.87
> 65	1.58 ± 0.15	11.59 ± 1.09	2.75 ± 0.28	26.60 ± 1.13	**5.03 ± 0.58**	15.32 ± 1.02
**Gender**						
Male	1.59 ± 0.16	10.95 ± 0.97	3.09 ± 0.23	25.52 ± 1.01	**5.12 ± 0.53**	13.94 ± 0.93
Female	1.50 ± 0.16	13.10 ± 1.18	2.47 ± 0.23	24.45 ± 1.16	3.29 ± 0.34**	15.01 ± 0.96
**Tobacco History**						
Smoker/Former-smoker	1.63 ± 0.16	10.71 ± 0.93	2.91 ± 0.21	26.69 ± 0.99	3.82 ± 0.40	15.15 ± 0.93
Non-smoker	1.24 ± 0.22	14.52 ± 2.10	2.94 ± 0.46	25.78 ± 2.15	4.20 ± 0.66	15.79 ± 1.87
**Histotypes**						
ADC	1.52 ± 0.15	13.31 ± 0.98	2.72 ± 0.20	25.58 ± 1.03	4.41 ± 0.43	14.17 ± 0.82
SqCC	1.55 ± 0.18	10.20 ± 1.23	3.01 ± 0.30	23.80 ± 1.20	4.14 ± 0.60	14.63 ± 1.26
LCC	1.86 ± 0.43	7.18 ± 2.62	2.57 ± 0.82	26.50 ± 2.97	4.07 ± 1.43	15.85 ± 2.86
**T stage**						
T1	1.19 ± 0.16	13.98 ± 1.67	2.46 ± 0.34	24.47 ± 1.56	3.72 ± 0.52	15.50 ± 1.41
T2	1.76 ± 0.17	11.74 ± 1.07	2.99 ± 0.25	26.15 ± 1.13	4.92 ± 0.54	13.00 ± 0.82
T3	1.23 ± 0.20	9.67 ± 1.46	2.50 ± 0.31	23.44 ± 1.46	3.63 ± 0.68	16.61 ± 1.91
T4	2.43 ± 0.69	12.05 ± 2.55	3.91 ± 0.28	22.83 ± 2.55	3.44 ± 0.74	14.98 ± 1.54
**N stage**						
N0	1.49 ± 0.13	12.31 ± 0.97	2.78 ± 0.20	25.79 ± 0.93	4.41 ± 0.43	**15.50 ± 0.88**
N1	1.50 ± 0.28	10.69 ± 1.51	2.80 ± 0.37	23.10 ± 1.52	4.41 ± 0.75	**13.40 ± 1.13**
N2	1.91 ± 0.35	11.84 ± 1.97	2.95 ± 0.46	24.30 ± 2.33	3.59 ± 0.75	10.85 ± 1.34*#
**Pathologic stage**						
I	1.35 ± 1.16	14.30 ± 1.38	2.83 ± 0.34	26.90 ± 1.45	4.31 ± 0.67	14.84 ± 1.27
II	1.53 ± 0.16	10.34 ± 1.08&##	2.60 ± 0.22	24.68 ± 1.12	4.63 ± 0.48	14.93 ± 0.98
IIIA	1.95 ± 032	11.47 ± 1.57	3.38 ± 0.32	23.00 ± 1.38	3.66 ± 0.59	12.65 ± 1.30
**Tumor size**						
≤3 cm	1.56 ± 0.19	**13.72 ± 1.27**	2.67 ± 0.25	25.43 ± 1.17	3.84 ± 0.42	14.51 ± 0.97
>3 cm	1.57 ± 0.14	10.65 ± 0.92*	2.90 ± 0.23	24.90 ± 1.03	4.64 ± 0.50	14.48 ± 0.93
**Relapse**						
No	1.60 ± 0.16	11.71 ± 0.95	3.02 ± 0.23	25.57 ± 1.05	4.21 ± 0.47	15.10 ± 0.98
Yes	1.55 ± 0.20	13.19 ± 1.75	2.50 ± 0.29	23.38 ± 1.47	4.40 ± 0.59	14.24 ± 1.19
**Adjuvant treatment**						
*Chemotherapy*						
No	1.56 ± 0.15	12.56 ± 1.10	2.69 ± 0.24	25.58 ± 1.06	4.75 ± 0.50	**15.68 ± 0.89**
Yes	1.51 ± 0.17	11.03 ± 1.07	2.94 ± 0.24	24.48 ± 1.16	3.57 ± 0.41&	12.71 ± 1.04*
*Radiotherapy*						
No	1.52 ± 0.14	12.00 ± 0.93	2.95 ± 0.20	24.89 ± 0.93	4.40 ± 0.41	14.78 ± 0.88
Yes	1.58 ± 0.19	11.70 ± 1.43	2.38 ± 0.28	25.72 ± 1.47	3.86 ± 0.57	13.50 ± 0.93

*P>0.05; **P>0.01; #N0 vs. N2, P=0.05; &P=0.07; ##Stage I vs. Stage II P=0.06

HS, heparan sulfate; CS, chondroitin sulfate; Col I, collagen type I; Col III, collagen type III, Col IV, collagen type IV; Col V, collagen type V; ADC, lung adenocarcinoma; SqCC: lung squamous cell carcinoma; LCC, large cell carcinoma.

Bolded values refer to a P-value with statistical significance (P<0.05).

When compared to stage I (P=0.06), we found a borderline significance between lower CS expression and tumor stage II, and between lower Col IV expression in patients who received chemotherapy (P=0.06). We did not find any statistical differences between clinicopathologic characteristics and HS, Col I, and Col III.

### 3.7 Prognostic value of morphometric variables

Out of 120 patients in our cohort, 65 progressed to death. In a univariable analysis (**Table 4**), OS for the entire cohort was significantly influenced by: gender (HR 0.39 for male versus female, CI 0.20-0.75, P=0.004), T stage (HR 2.69 for T3-T4 versus T1-T2, CI 1.46 – 4.94, P=0.001), tumor size (HR 1.95 for > 3cm versus ≤ 3cm, CI 1.08-3.53, P=0.026), metastases (HR 2.98 for present versus absent, CI 1.60-5.53, P=0.001), and radiotherapy (HR 0.40 for No versus Yes, CI 0.21-0.76, P=0.005). We also observed that high SPARC and WNT3A expressions in the tumor stroma had a significant influence on OS (HR 0.55 for higher versus low expression, CI 0.30-1.00, P=0.050; HR 0.54 for higher versus lower expression, CI 0.30–0.99, P=0.046; respectively).

**Table 4:** Variables associated with overall survival in 120 non-small cell lung cancer patients.

Variables	Mean OS (months)	Univariate Analysis^b^			Multivariate Analysis^c^	
		HR (95% CI)	HR	P value	HR (95% CI)	P value
**Age (years, median)**						
≤ 65 (reference)	74					
> 65	82	0.96 (0.54-1.71)	-0.039	0.895		
**Gender**						
Male (reference)	59					
Female	107	0.39 (0.20-0.75)	-0.942	**0.004**	0.39(0.17-0.90)	**0.028**
Smoker Status^a^						
Non-Smoker (reference)	95					
Smoker/Former-smoker	84	1.38(0.67-2.89)	0.327	0.382		
**Histological type**						
Adenocarcinoma (reference)	83			0.245		
Squamous cell carcinoma	74	1.65(0.89-3.09)	0.498	0.120		
Large cell carcinoma	60	1.88(0.45-7.94)	0.633	0.389		
**T stage†**						
T1+T2 (reference)	97					
T3+T4	45	2.69(1.46-4.94)	0.989	**0.001**	2.14(1.03-4.74)	**0.041**
**N stage†**						
N0 (reference)	85			0.497		
N1	55	1.63(0.71-3.70)	0.486	0.247		
N2	75	0.992(0.45-2.16)	-0.008	0.983		
**Pathological Stage†**						
I+II (reference)	87					
III	66	1.35(0.71-2.58)	0.301	0.362		
**Tumor Size**						
≤ 3 cm (reference)	100					
> 3 cm	61	1.95(1.08-3.53)	0.669	**0.026**	3.65(1.58-8.40)	**0.002**
Relapse^a^						
Absent (reference)	99					
Present	48	2.98(1.60-5.53)	1.091	**0.001**	2.65(1.27-5.51)	**0.009**
**Adjuvant therapy**						
Chemotherapy ^a^						
Yes (reference)	64					
No	91	0.62(0.35-1.11)	-0.469	0.110	4.61(1.74-12.20)	**0.002**
Radiotherapy ^a^						
Yes (reference)	45					
No	91	0.40(0.21-0.76)	-0.906	**0.005**	0.22(0.08-0.59)	**0.003**
**Protein expression** (≤ mean vs > mean)						
*Heparan Sulfate* ^a^						
≤ 1.55% (reference)	72					
> 1.55%	91	0.77(0.42-1.41)	-0.258	0.402	0.44(0.19-1.02)	**0.055**
***Chondroitin Sulfate***						
≤ 11.92% (reference)	85					
> 11.92%	74	1.02(0.57-1.82)	0.022	0.941		
***Col I***						
≤ 2.81% (reference)	78					
> 2.81%	78	1.12(0.62-2.01)	0.114	0.703		
***Col III***						
≤ 25.04% (reference)	81					
> 25.04%	77	0.92(0.52-1.64)	-0.080	0.786		
***Col IV***						
≤ 4.30% (reference)	86					
> 4.30%	67	1.30(0.70-2.42)	0.264	0.405		
***Col V***						
≤ 14.42% (reference)	72					
> 14.42%	87	0.83(0.46-1.49)	-0.184	0.535		
***E-cadherin***						
≤ 23.99% (reference)	91					
> 23.99%	71	1.35(0.75-2.44)	0.300	0.320		
*β-catenin* ^a^						
≤ 21.25% (reference)	87					
> 21.25%	77	1.08(0.60-1.93)	0.076	0.799		
*WNT1* ^a^						
≤ 13.55% (reference)	85					
> 13.55%	73	1.11(0.62-2.00)	0.108	0.715		
*WNT3A* ^a^						
≤ 19.17% (reference)	61					
> 19.17%	97	0.54(0.30-0.99)	-0.612	**0.046**	1.44(0.69-3.01)	0.334
***WNT5A***						
≤ 42.48% (reference)	88					
> 42.48%	68	1.36(0.73-2.51)	0.306	0.329	0.768(0.35-1.66)	0.501
***WNT5B***						
≤ 3.75% (reference)	80					
> 3.75%	77	0.97(0.54-1.73)	-0.034	0.908		
***SPARC***						
≤ 14.25% (reference)	65					
> 14.25%	98	0.55(0.30-1.00)	-0.601	**0.050**	0.31(0.13-0.75)	**0.009**

a Some cases had missing follow-up information: Smoke status (31); Tumor size (2); Relapse (21); Chemotherapy (4); Radiotherapy (4); Status (21); Heparan Sulfate (1); β-catenin (2); WNT1 (2); WNT3A (2).

b Univariate analysis was carried out without any adjustment in order to generate hazard ratios with confidence intervals for individual risk for each of the parameters on survival.

c Multivariate analysis was carried out to analyze the effects of several risk parameters on survival.

† According to the 8th Edition International Association for the Study of Lung Cancer (21).

OS, Overall Survival; HR, Hazard Ratio (β coefficient); CI, Confidence Interval; Col I, collagen type I; Col III, collagen type III, Col IV, collagen type IV; Col V, collagen type V.

Bolded values refer to a P-value with statistical significance (P<0.05). The univariate and multivariate analysis employed a Cox proportional hazards model (Chi-square 33.223; P<0.001).

Conversely, in a multivariable analysis, gender, T stage, tumor size, metastases, adjuvant therapy, and SPARC were significantly associated with OS, whereas high HS expression had only a borderline association with OS (P=0.055). WNT3A and WNT5A were co-variables in this mathematical model (Chi-square 33.223; P<0.001). Mean OS was 97 months for patients with SPARC expression >14.25% compared to 65 months for patients with expression ≤14.25% (**Supplementary Figure 3**).

### 3.8 Validation of study cohort by *in silico* data mining

#### 3.8.1 mRNA and protein expression

To create a possibility of the comparison between our data, normal samples, and other results, we used the UALCAN to analyze the TCGA database and to obtain levels of mRNA expression of our markers of interest, except the GAGs, in ADC and SqCC. The database did not include data for LCC.

Compared to normal tissues, in ADC (**Supplementary Figure 4**) the mRNA expression level of E-cadherin, β-catenin, Col I, Col III, Col V, WNT1, and WNT5B showed significant upregulation upregulated (P<0.01, for all), whereas WNT3A mRNA expression levels showed significant downregulation (P<0.01).

For SqCC (**Supplementary Figure 5**), E-cadherin, Col I, Col III, Col V, WNT5A, and WNT5B mRNA expression levels showed significant upregulation (P<0.01, for all), and only WNT3A mRNA expression levels showed significant downregulation (P<0.01) when compared to the normal tissue.

We used the same platform to analyze protein expression data from the Clinical Proteomic Tumor Analysis Consortium (CPTAC). Until the time of this study, the consortium database only covered ADC samples but included data on most of the molecules explored in our study, except for WNT1, WNT3A, and WNT5B. Compared to normal tissues, E-cadherin, Col I, Col III, Col V, WNT5A, and SPARC showed significant overexpression (P<0.01, except SPARC, P=0.02; **Supplementary Figure 6**), whereas β-catenin, HS, CS, and Col IV showed significant under-expression (P<0.01; **Supplementary Figure 6**).

Consistent with our data, the results of E-cadherin, Col I, Col III, Col V, WNT5A, and SPARC proteins were overexpressed in ADC, whereas β-catenin, HS, CS, and Col IV were under-expressed.

#### 3.8.2 Association between expression and clinicopathological parameters

Using the TCGA (Pan Cancer Atlas) data, we collected data from the cBio Cancer Genomics Portal which included clinical data of 939 patients with ADC and SqCC in pathological stages I to IIIA, like our cohort. We also collected the mRNA expression of all our markers of interest for this same group of individuals.

When correlating this data, we noted the association between gender and β-catenin, WNT1, WNT3A, WNT5A, and WNT5B expression; and a significant difference between histotypes and β-catenin, WNT1, WNT3A, WNT5A, and WNT5B expression. We also observed a significant difference between the expression of WNT1 and T2 stage and N1 stage. In patients who developed metastasis during follow-up, we observed a significant difference between the expression of β-catenin, WNT3A, and WNT5A. We did not find a statistical difference between other clinicopathologic characteristics and the expression of these markers (**Supplementary Table 2**).

Using the same analysis for the stoma markers, we observed a significant difference between the histotypes and the expression of Col III. We did not find significant differences between any other clinicopathologic characteristic and the expression of these markers (**Supplementary Table 3**).

#### 3.8.3 Prognostic value GAGs, SPARC, EMT and collagen types

We used a Kaplan-Meier plotter analysis to find the correlation between expression levels and OS in NSCLC patients for all makers analyzed in our study (**Supplementary Figure 7**).

We set the cutoff for high or low expression using the group median expression. As shown in **Supplementary Figure 7**, patients with high Col IV, WNT3A, WNT5A, WNT5B, and SPARC expression had longer OS (P<0.05). On the other hand, low Col I and WNT1 expression correlated with longer OS (P ≤ 0.05). We found no statistical significance effect of E-cadherin, β-catenin, HS, CS, Col III, and Col V on OS.

#### 3.8.4 Biologic interaction among GAGs, SPARC, EMT and collagen types

Given the above, our next step was to conduct a functional enrichment analysis using the STRING database, a search tool for protein interaction, to find a significant protein-protein interaction (PPI) network. **Figure 11** shows the molecular organization of this network. The network is made of differentially connected nodes, each node represents a protein, and the edges represent their dynamic interactions. The PPI enrichment P-value was <1.0e-16. This shows that these proteins are at least partially biologically connected as a group.

We also performed an analysis in Metascape to assess the function and the biological process of the genes corresponding to the proteins of our interest. **Supplementary Figure 8A** shows the heatmap of enriched terms across input gene lists. The main terms we observed were: “Epithelial to mesenchymal transition in colorectal cancer”, “ECM proteoglycans”, and “proteoglycans in cancer”. **Supplementary Figure 8B** shows the network formed by these enriched terms. **Supplementary Figure 8C** shows the top-level Gene Ontology biological processes. The most statistically significant terms within this group were “development process”, “signaling”, “response to stimulus”, and “cellular process”.

Taken together, these *in silico* results suggest a strong integration between our proteins of interest and fundamental cellular processes in carcinogenesis, which confirms our experimental results.

## 4 Discussion

Under the scenario of our study cohort, the locoregional and distant metastases not previewed by TNM stage and histological classification are the possible reasons for surgical resection failures at curing some early-stage NSCLC patients. The query of interest is whether ancillary information gathered from either the tumor cells or its tumor stroma can help us to improve risk stratification and patient selection for adjuvant systemic treatment. The development of cancer cell invasion and metastases certainly encompasses a series of complex and sequential stages. Among them are the EMT, loss of basement membranes, and remodeling of the interstitial extracellular matrix barriers by tumor cells. These processes are considered important because the tumor-reprogrammed lung microenvironment promotes both primary lung tumors and metastasis by contributing mainly to mechanical and functional barriers (4). The loss of these barriers facilitates the migration of tumor cells and penetration of tumor by blood vessels (34–37). Other important glycoproteins present in lung cancer are the SPARC and WNTs. These glycoproteins act on the remodeling of the extracellular matrix and the EMT and provide tumor growth and metastasis (20, 38–40).

Therefore, to understand the relationship between EMT, matricellular barriers, and the metastatic process, we used a step-stage design. We first used IHC, TEM, and IF to characterize EMT proteins, glycosaminoglycans, collagen types, SPARC, and WNT proteins in ADC, SqCC, and LCC histological subtypes. We then examined the clinical association between these markers and the data of 120 patients with surgically excised NSCLC. Afterwards, we analyzed the impact of these markers on patients’ survival. Lastly, we validated the study cohort using *in silico* data mining. While one of the major limitations of our study is the small number of NSCLC cases used, the data obtained using IHC, IF and TEM and the image analysis applied minimized this limitation. Thus, we provide new evidence that NSCLC cells can express EMT and matricellular proteins with known mechanical barrier function. Expression of those proteins is associated both with the Wnt pathway and with significantly longer overall patient survival. We also found that there is strong integration between our proteins of interest, their expression/behavior is like what we obtained in this work, and they act on fundamental cellular processes in carcinogenesis.

While most of the studies evaluated NSCLC progression by either loss of tumor suppressor genes and/or activation of oncogenes (41), we described NSCLC progression during the EMT phenotypic changes in rendering tumor cells invasive and able to metastasize distant organs. Another main innovative feature of our study was the description of the relationship between EMT-ECM components-Wnt signal pathway with histological subtypes, TNM stage, and survival. Throughout our study, we showed that, during the EMT process, the low expression of E-cadherin and B-catenin created a poor tumor portion barrier against tumor invasion. Wnt signaling, mainly by WNT5A, and SPARC enhanced this barrier and facilitated tumor progression.

We also found that tumor-associated GAGs and collagen mechanical barriers reinforced the functional barriers between EMT, WNT proteins and malignant cells. The collagen mechanical barriers correspond to different levels of HS, CS, and collagen fibers, which are reorganized to locate and characterize malignant cells. In this scenario, we inferred that CS, Col III, and Col V also have a high chance to create a mechanical barrier against malignant cells and prevent the invasion of the interstitial ECM. Importantly, the high expression of WNT1, CS, and Col V was associated with tumors in stages I, and N0-N1. These findings suggest that increased expression of these markers occurs at early stages of carcinogenesis, which decreases with tumor growth. Therefore, these markers emerge as promising for therapeutic decisions before surgery. In addition, we also observed the influence, direct and indirect, of WNT3A, WNT5A, HS, and SPARC on the overall survival of patients with early-stage NSCLC. This influence shows that there is a relevant regulation between these components in tumor progression. However, there are some major points which need to be addressed, as discussed below.

Firstly, the issue to be addressed is the significance of EMT-like phenotypic changes for the interaction between collagen matrix and malignant cells. We observed that the scattering intensity was higher in EMT-positive malignant cells from SqCC and ADC compared to LCC, to promote ECM invasion by individual malignant cells, in case of LCC, and by cells groups in case of ADC and SqCC. Thus, these findings describe a partial EMT – e.g., a hybrid EMT (42, 43) – as the predominant, hierarchical immune phenotype in SqCC and ADC of the lung compared to LCC. This suggests the emerging notion that a partial EMT, but not necessarily a complete EMT, is associated with aggressive tumor progression (44). As recently reported, there is no mesenchymal transition in a hybrid EMT, as the tumor cells retain E-cadherin (45). This could explain the high expression of E-cadherin found in our work, mostly in SqCC and ADC. We also observed a strong correlation between TEM proteins and WNT5A. WNT5A can interact with the tumor, functioning as both a suppressor and a promoter (46, 47). When acting on cell adhesion, motility, and cell polarity, WNT5A interacts with intracellular effectors through the Wnt signaling pathway and with ECM structures. Thus, it acts in different ways on the process of EMT and on the B-catenin/E-cadherin complex (48, 49).

Moreover, these EMT modifications resulted from the dissemination of cancer and invasion of ECM, leading to changes in GAGs and fibril structural organization of collagens visualized at IF and TEM These changes then caused the endogenous GAGs/collagen to degenerate and the emergence of new GAGs/collagen of a diminished structural organization, as previously described in breast cancer (50). In addition, at TEM, we demonstrated appreciably larger cell-cell boundaries, suggesting abnormal adherent junctions because of cancer propagation into the interstitial ECM. As mentioned, the tissue loses cell-cell adhesion, causing diminished, long-range intermolecular bonding rigidity to Col I, Col III and Col V, with the disruption of collagen fibrils structure as well as HS/CS. Previous studies described that some cancer cells can produce collagen types I, III, IV, V and VI (51–53). The alteration in the deposition of these fibrillar collagens alters the rigidity of the tumor matrix and may contribute to the metastatic behavior of tumor cells, mediated, for example, by cancer-associated fibroblasts (54). Besides, tissue remodeling is a crucial step during carcinogenesis, a transformation of epithelial cells being associated with metalloproteinases in collagen degradation by synthesis of fibrillary and non-fibrillary matrix proteins (55).

In our cohort, we noted a high expression of Col III and V, and a low expression of Col I and IV. We observed the same increased expression of Col III in pancreatic cancer tissue (56). Concerning the Col I expression, it has been proposed that the interaction of epithelial cells with Col I contributes to increased cell motility accompanying EMT, critical in disease progression (57). In colorectal cancer, Col I was described to downregulate the E-cadherin and B-catenin expression (58). However, we did not observe a correlation between Col I and ETM proteins. The expression of these markers in our study was inverse, suggesting a possible response to changes in ECM. In addition, the reduction of the structural assembly, and consequent decrease in expression of HS, and Col I and IV within ECM, can be associated with the activity of collagenases, a group of collagen-degrading enzymes (59). Furthermore, not only there was a change in the concentration of fibrillar collagens, but also in the levels of immunoreactive GAGs, such as HS and CS, and their proteoglycans. This change can alter the stiffness of the ECM and participate in the EMT process (60, 61). Although these compounds do not alter the biomechanical characteristics of ECM, they can play an important role in tumor ECM remodeling (60).

In the current study, it also became evident that, at the supramolecular level changes in collagen in cancer-invaded ECM, there was an association, direct or indirect, with WNT1, WNT3A, and WNT5A signaling. This paved the way for NSCLC classification and improved understanding of mechanisms of cancer growth. These findings can be confirmed by recent studies that have shown the Wnt/β-catenin signaling pathway integrates signals from other proteins and signaling pathways, such as the possibility of the pathway being modulated by integrins (62). These studies suggest that this modulation process can be done through the expression of WNT ligands, receptors, and inhibitors, or through the modulation of β-catenin concentration in different cell types (63).

We also highlight that we found an important association between HS with SPARC and WNT3A. In tumor stroma, the composition of the ECM and the population of cells present there are quite different when compared to normal tissue. As the tumor develops, a series of processes also occur that remodel the stromal tissue to regulate tumor progression. In general, several glycoproteins, collagens, GAGs, proteoglycans, and many other proteins that promote cell proliferation and motility drive this modification process in the ECM (64–66). Among the GAGs, HS, when cleaved by heparanase, alters its structure and function, and contributes to tumor-mediated remodeling of both cell surfaces and the ECM (67–69). It is also known that HS proteoglycans extracellularly regulate WNT signaling, including WNT3A (70). Thus, these activities increase the bioavailability of HS-linked growth factors (71) that recruit metastatic malignant cells, and support their survival and growth, thus driving the metastatic process. Previous studies report that HS plays a crucial role in cell proliferation and metastasis in breast cancer (72), rhabdomyosarcoma (73), and NSCLC (74). Furthermore, changes in the microenvironment also affect the expression and function of other molecules, such as SPARC. This is a matricellular glycoprotein that directly participates in the ECM remodeling process, regulating processes, such as metalloproteinase secretion and cell-matrix interactions (75). Recent studies showed that SPARC favored the migration and invasion of endometrial carcinoma cells *in vitro* and *in vivo* (76).

We also found that CS was correlated with WNT1. CS is a transmembrane glycoprotein with a large extracellular domain and a short intracellular domain (77). Its extracellular domain includes subdomains that can interact with various components of the ECM, such as Col V and Col VI. Thus, they promote the activation of oncogenic pathways, growth factors, and EMT, increasing the migration of malignant cells (78–80). As it is widely accepted, GAGs can shape morphogenesis gradients and modulate morphogenesis signaling through their binding affinities with a variety of signaling molecules due to their various structures (81). Furthermore, as we could observe in this study, an increase in CS expression in tissues has been described in several tumor types (82–85). Thus, it can be suggested that this increase could influence ECM remodeling (as previously mentioned for HS), altering the tumor microenvironment and modifying cell-signaling processes. Even though we failed to locate any other studies that demonstrate this direct affinity between CS and WNT1, our finding may be supported by the study that showed that CS-E (a CS with an increased level of 4,6-O-disulfated disaccharides) inhibited the Wnt signaling pathway *in vitro* assays using breast cancer cells (86).

Another important issue to address is the impact of the morphometric variables on clinicopathologic features. Histologically, we observed that NSCLC encompassed a bimodal spectrum of malignancies. On one side, there are ADC and SqCC subtypes, both characterized by EMT process, associated with considerable desmoplasia due to accumulation of ECM components, which were closely associated with basement membrane invasion by groups of cells at TEM. On the opposite side, there is LCC, a NSCLC subtype composed by malignant cells immersed in a poor desmoplastic stroma with low EMT process, and low levels of basement membrane and interstitial ECM components, associated with invasion by isolated cells at TEM. Clinically, we found that younger patients presented more small-sized aggressive tumors, LCC-histotype with low expression of E-cadherin and Col IV, and high expression of WNT1 and WNT5B. Moreover, large tumors (bigger than 3.0 cm) showed low expression of CS and higher expression of WNT5A. Notably, the high expression of WNT1, CS and Col V was associated with tumors in stage I and II, and N0-N1, suggesting that increased expression of these markers occurs at very earlier stages of carcinogenesis. Considering the findings discussed so far, it is possible to see that, sometimes, the molecules studied in the microenvironment here presented may not act directly on the clinical characteristics, affecting the aggressiveness of the tumor. However, the observed behavior is very consistent with the remodeling of this environment for progression and metastasis. This makes us speculate that the study of these compounds is important for the analysis of early-stage patients regarding progression and the intention to prevent operated patients from having relapses due to possible occult metastasis.

We observed a consistent result in our Cox regression analysis. We performed this analysis to examine the impact of morphometric variables on survival. We observed that OS, for the entire cohort, was significantly influenced by gender, T stage, tumor size, metastases, and radiotherapy. We also observed a significant influence for better OS by higher SPARC and WNT3A expression in tumor stroma. In multivariable analysis, there was a significant association between gender, T stage, tumor size, metastasis, adjuvant therapy, and SPARC and OS. High expression of HS presented borderline influence for OS. WNT3A and WNT5A were co-variables in this mathematical model. Mean OS was 97 months for patients with SPARC expression >14.25% compared to 65 months for patients with expression ≤14.25%. These findings are consistent with the literature, in which there is an association between low SPARC expression and a worse prognosis in endometrial carcinoma (76), colorectal cancer (87, 88), and NSCLC (89). Furthermore, it is described that, depending on the tumor microenvironment, SPARC can act both as a tumor suppressor and as an oncogene (76). Thus, we speculate that the HS and WNT proteins, which influenced our regression model, may affect these questions about the paradoxical effect of SPARC. Since HS proteoglycans modulate WNT signaling, HS modifications influence disease progression (90–92). In addition, we have the entire ECM modification process discussed previously.

To complete these results of the roles of E-cadherin, β-catenin, Col I, Col III, Col V, WNT1, and WNT5B in NSCLC, we explored, *in silico*, whether the mRNA level of these proteins related to clinical outcome of the patients. We confirmed that in ADC and SqCC there was a significant upregulation of the mRNA expression levels of E-cadherin, β-catenin, Col I, Col III, Col V, WNT1, and WNT5B, while there was a significant downregulation of Wnt3A mRNA expression. By Clinical Proteomic Tumor Analysis Consortium (CPTAC) data for protein expression only available for ADC, we confirmed that there was a significant overexpression of E-cadherin, Col I, Col III, Col V, WNT5A, and SPARC protein, while there was a significant under-expression of β-catenin, HS, CS, and Col IV. The behavior of this protein expression was similar in our study, proving the consistency of our results. As for clinicopathological characteristics, we observed some correlations between β-catenin, WNT proteins, and histological subtypes (ADC and SqCC), which we observed only with β-catenin and WNT5A in our study. However, except for WNT1 expression and T and N stages, the data also showed no significant correlations with pathological stage or TNM as in our study. Kaplan-Meier curves showed that patients with higher Col IV, WNT3A, WNT5A, WNT5B, and SPARC expression had a longer OS, whereas patients with higher Col I and WNT1 expression had a **s**horter OS. The function and the biological process of the genes corresponding to the proteins of our interest showed that the top-level Gene Ontology biological processes involved were “development process”, “signaling”, “response to stimulus”, and “cellular process”.

In summary, the data presented provide important hierarchical evidence that genes and proteins associated with EMT, WNT signal pathway, and ECM are involved in the proliferative signal of cancer cells, spaced desmosomes, and facilitating cell motility. This evidence suggests sequential steps for primary tumor invasion and metastasis in patients that were in early-stages and who underwent surgical resection. Importantly, this study indicates that NSCLC with increased expression of mechanical barrier proteins and low expression of the functional proliferative barrier presents a low risk of patient mortality due to metastasis and promising new therapeutic targets. In addition, mechanistic insight into the major findings needs to be complemented with *in vitro* data. Therefore, in this emerging scenario of personalized treatments, future studies are necessary to include such observations in the clinic, as the basis of a biomarker measured in circulation and/or urine for selecting patients who may benefit from these.

## Data availability statement

The datasets used and/or analyzed during the current study are available from the corresponding author on reasonable request.

## Ethics statement

The studies involving human participants were reviewed and approved by Ethics Committee of the Faculty of Medicine, University of São Paulo (protocol number 150.443/2019). Written informed consent for participation was not required for this study in accordance with the national legislation and the institutional requirements.

## Author contributions

Conception and design: VC and CB. Writing, review, and editing: VC, CB, and TP. Data analysis and interpretation: VC, CB, TP. Investigation: CB, TP, JM-R, JM, LS, AV, and WT. Provision of study materials or patients: AA and TT. Supervision: VC. Administrative support: VC. All authors contributed to the article and approved the submitted version.

## Funding

This work was supported by Sao Paulo Research Foundation (FAPESP; 2018/20403-6), the National Council for Scientific and Technological Development (CNPq 303735/2021-0), and Coordenação de Aperfeiçoamento de Pessoal de Nível Superior - Brasil (CAPES; Finance Code 001).

## Acknowledgments

We are grateful to Ms. Esmeralda Miristeni Eher and Ms. Sandra de Morais Fernezlian for their expertise in immunohistochemical protocols, and to Aline Assato for her expertise to construct of the TMAs slides.

## Conflict of interest

The authors declare that the research was conducted in the absence of any commercial or financial relationships that could be construed as a potential conflict of interest.

## Publisher’s note

All claims expressed in this article are solely those of the authors and do not necessarily represent those of their affiliated organizations, or those of the publisher, the editors and the reviewers. Any product that may be evaluated in this article, or claim that may be made by its manufacturer, is not guaranteed or endorsed by the publisher.

## Supplementary material

Supplementary Figure 1 Negative controls of immunofluorescence for E-cadherin and β-catenin in the different histological subtypes of NSCLC. The stained nuclei are represented in blue (DAPI). Original magnification: 40X. LCC, large cell carcinoma; ADC, lung adenocarcinoma; SqCC: lung squamous cell carcinoma.

Supplementary Figure 2 Negative controls of immunofluorescence for chondroitin sulfate and heparan sulfate (green), and collagen types I, III, IV, and V (red). The stained nuclei are represented in blue (DAPI). Original magnification: 40X.

Supplementary Figure 3 Survival curve for SPARC expression. The blue line represents the high expression of SPARC, and the yellow line the low expression of SPARC.

Supplementary Figure 4 mRNA expression of the interest markers in lung adenocarcinoma from data obtained from “The Cancer Genome Atlas” (TCGA). **P<0.01. ADC, lung adenocarcinoma; Col, collagen type.

Supplementary Figure 5 mRNA expression of the interest markers in lung squamous cell carcinoma from data obtained from “The Cancer Genome Atlas” (TCGA). **P<0.01. SqCC: lung squamous cell carcinoma; Col, collagen type.

Supplementary Figure 6 Expression of the interest proteins in adenocarcinoma from data obtained from the Clinical Proteomic Tumor Analysis Consortium (CPTAC). *P<0.05, **P<0.01. ADC, lung adenocarcinoma; HS, heparan sulfate; CS, chondroitin sulfate; Col, collagen type.

Supplementary Figure 7 Kaplan Meier curves for our interest markers obtained in KM-Plotter data base. The black line represents a low expression of the marker and the red line, the high expression.

Supplementary Figure 8 Metascape enrichment analysis. **(A)** Heatmap of enriched terms across input gene lists. **(B)** Network formed by these enriched terms. **(C)** Biological processes were involved the gene list colored according to the P-value.
