# Corrigendum: Modeling extracellular matrix through histo-molecular gradient in NSCLC for clinical decisions

**DOI:** 10.3389/fonc.2024.1543813

**Published:** 2025-01-09

**Authors:** Camila Machado Baldavira, Tabatha Gutierrez Prieto, Juliana Machado-Rugolo, Jurandir Tomaz de Miranda, Lizandre Keren Ramos da Silveira, Ana Paula Pereira Velosa, Walcy Rosolia Teodoro, Alexandre Ab’Saber, Teresa Takagaki, Vera Luiza Capelozzi

**Affiliations:** ^1^ Department of Pathology, Faculty of Medicine, University of São Paulo, São Paulo, Brazil; ^2^ Health Technology Assessment Center, Clinical Hospital, Medical School of São Paulo State University, Botucatu, São Paulo, Brazil; ^3^ Rheumatology Division of the Hospital das Clínicas da Faculdade de Medicina da Universidade de São Paulo, Faculty of Medicine, University of São Paulo, São Paulo, SP, Brazil; ^4^ Division of Pneumology, Instituto do Coração (Incor), University of São Paulo Medical School (USP), São Paulo, Brazil

**Keywords:** lung cancer, extracellular matrix, epithelial-to-mesenchymal transition, WNT signaling pathway, glycosaminoglycans

In the published article, an author name was incorrectly written as “Lizandre Keren Ramos de Oliveira”. The correct spelling is “Lizandre Keren Ramos da Silveira”.

In the published article, the initials used for Lizandre Keren Ramos da Silveir in the **Author contributions** section was incorrectly written as “LO”. The correct spelling is “LS”.

In the published article, there was an error in the **Data availability statement**.

The EMPIAR repository has informed us that the transmission electron microscopy images we submitted for deposit will be removed from their database, as they do not store this specific type of microscopy image. We had deposited these images as per the journal’s request when we submitted the article, but unfortunately, this issue has arisen. As the corresponding author, I searched for alternative repositories but was unable to find any suitable options. Nevertheless, in compliance with ethical research standards and best practices, we, the authors, are fully committed to providing any data or analyses conducted during the study upon request from our peers.

“The datasets presented in this study can be found in online repositories. The names of the repository/repositories and accession number(s) can be found below: EMPIAR, accession number EMPIAR-11226.”

The correct **Data availability statement** appears below.

“The datasets used and/or analyzed during the current study are available from the corresponding author on reasonable request.”

The authors apologize for these errors and state that this does not change the scientific conclusions of the article in any way. The original article has been updated.

