# Supplementary material for: Modeling extracellular matrix through histo-molecular gradient in NSCLC for clinical decisions
Source: Front Oncol. 2022 Nov 14;12:1042766. doi: 10.3389/fonc.2022.1042766 (PMC9703002; doi:10.3389/fonc.2022.1042766)
Supplement: Supplementary Figure 1 — Negative controls of immunofluorescence for E-cadherin and β-catenin in the different histological subtypes of NSCLC. The stained nuclei are represented in blue (DAPI). Original magnification: 40X. LCC, large cell carcinoma; ADC, lung adenocarcinoma; SqCC: lung squamous cell carcinoma. [file DataSheet_1.zip › Table 1.DOCX]

**Supplementary Table 1.** Association between NSCLC histological subtypes and the morphometric variables (Pearson’s Chi-square;P<0.05)

|  | **Histological Subtype** | | |  |
| --- | --- | --- | --- | --- |
| **Protein expression** | **ADC** | **SqCC** | **LCC** | **P value** |
| ***Heparan Sulfate ^a^*** |  |  |  | 0.215 |
| ≤ 1.55% | 45 (37.8%) | 23 (19.3%) | 2 (1.7%) |  |
| > 1.55% | 27 (22.7%) | 17 (14.3%) | 5 (4.2%) |  |
| ***Chondroitin Sulfate*** |  |  |  | 0.307 |
| ≤ 11.92% | 38 (31.7%) | 26 (21.7%) | 5 (4.2%) |  |
| > 11.92% | 35 (29.2%) | 14 (11.7%) | 2 (1.7%) |  |
| ***Col I*** |  |  |  | 0.123 |
| ≤ 2.81% | 45 (37.5%) | 17 (14.2%) | 3 (2.5%) |  |
| > 2.81% | 28 (23.3%) | 23 (19.2%) | 4 (3.3%) |  |
| ***Col III*** |  |  |  | 0.175 |
| ≤ 25.04% | 40 (33.3%) | 26 (21.7%) | 2 (1.7%) |  |
| > 25.04% | 33 (27.5%) | 14 (11.7%) | 5 (4.2%) |  |
| ***Col IV*** |  |  |  | 0.857 |
| ≤ 4.30% | 44 (36.7%) | 26 (21.7%) | 4 (3.3%) |  |
| > 4.30% | 29 (24.2%) | 14 (11.7%) | 3 (2.5%) |  |
| ***Col V*** |  |  |  | 0.089 |
| ≤ 14.42% | 49 (40.8%) | 22 (18.3%) | 2 (1.7%) |  |
| > 14.42% | 24 (20.0%) | 18 (15.0%) | 5 (4.2%) |  |
| ***E-cadherin*** |  |  |  | 0.474 |
| ≤ 23.99% | 43 (35.8%) | 20 (16.7%) | 5 (4.2%) |  |
| > 23.99% | 30 (25.0%) | 20 (16.7%) | 2 (1.7%) |  |
| ***β-catenin*** |  |  |  | **0.002** |
| ≤ 21.25% | 48 (40.7%) | 14 (11.9%) | 6 (5.1%) |  |
| > 21.25% | 24 (20.3%) | 25 (21.2%) | 1 (0.8%) |  |
| ***WNT1*** |  |  |  | 0.165 |
| ≤ 13.55% | 43 (36.4%) | 26 (22.0%) | 2 (1.7%) |  |
| > 13.55% | 29 (24.6%) | 13 (11.0%) | 5 (4.2%) |  |
| ***WNT3A*** |  |  |  | 0.387 |
| ≤ 19.17% | 40 (33.9%) | 20 (16.9%) | 2 (1.7%) |  |
| > 19.17% | 32 (27.1%) | 19 (16.1%) | 5 (4.2%) |  |
| ***WNT5A*** |  |  |  | **0.002** |
| ≤ 42.48% | 46 (38.3%) | 13 (10.8%) | 6 (5.1%) |  |
| > 42.48% | 27 (22.5%) | 27 (22.5%) | 1 (0.8%) |  |
| ***WNT5B*** |  |  |  | 0.258 |
| ≤ 3.75% | 44 (36.7%) | 24 (20.0%) | 2 (1.7%) |  |
| > 3.75% | 29 (24.2%) | 16 (13.3%) | 5 (4.2%) |  |
| ***SPARC*** |  |  |  | 0.966 |
| ≤ 14.25% | 42 (35.0%) | 24 (20.0%) | 4 (3.3%) |  |
| > 14.25% | 31 (25.8%) | 16 (13.3%) | 3 (2.5%) |  |

^a^ Some cases has missing data: Heparan Sulfate (1); β-catenin (2); WNT1 (2); WNT3A (2).

**Abbreviations:** ADC, adenocarcinoma; SqCC, squamous cell carcinoma; LCC, large cell carcinoma; Col I, collagen type I; Col III, collagen type III, Col IV, collagen type IV; Col V, collagen type V.
