# Supplementary material for: Modeling extracellular matrix through histo-molecular gradient in NSCLC for clinical decisions
Source: Front Oncol. 2022 Nov 14;12:1042766. doi: 10.3389/fonc.2022.1042766 (PMC9703002; doi:10.3389/fonc.2022.1042766)
Supplement: Supplementary Figure 1 — Negative controls of immunofluorescence for E-cadherin and β-catenin in the different histological subtypes of NSCLC. The stained nuclei are represented in blue (DAPI). Original magnification: 40X. LCC, large cell carcinoma; ADC, lung adenocarcinoma; SqCC: lung squamous cell carcinoma. [file DataSheet_1.zip › Table 2.DOCX]

**Supplementary Table 2.** Clinicopathologic characteristics and mean mRNA expression of E-cadherin, β-catenin, WNTs signaling proteins, and SPARC (TCGA database, N=939; Chi-square test, P<0.05)

| **Characteristics** | **E-cadherin** | | | **P** | **β-catenin** | | | **P** | **WNT1** | | **P** | **WNT3A** | | | **P** | **WNT5A** | | | **P** | **WNT5B** | | | **P** | **SPARC** | | | **P** |
| --- | --- | --- | --- | --- | --- | --- | --- | --- | --- | --- | --- | --- | --- | --- | --- | --- | --- | --- | --- | --- | --- | --- | --- | --- | --- | --- | --- |
|  | **Low** | **High** | |  | **Low** | **High** | |  | **Low** | **High** |  | **Low** | **High** | |  | **Low** | **High** | |  | **Low** | **High** | |  | **Low** | **High** | |  |
| **Age (years)^a^** |  | | | 0.98 |  | | | 0.13 |  | | 0.79 |  | | | 0.08 |  | | | 0.62 |  | | | 0.15 |  | | | 0.24 |
| ≤65 | 21.7% | | 21.4% |  | 20.5% | | 22.6% |  | 21.4% | 21.7% |  | 22.9% | | 20.2% |  | 21.7% | | 21.4% |  | 22.6% | | 20.5% |  | 22.4% | | 20.7% |  |
| >65 | 28.6% | | 28.3% |  | 29.9% | | 26.9% |  | 28.7% | 28.2% |  | 26.9% | | 29.9% |  | 27.7% | | 29.2% |  | 27.1% | | 29.8% |  | 27.3% | | 29.6% |  |
| **Gender** |  | | | 0.69 |  | | | **0.002** |  | | **0.01** |  | | | **<0.001** |  | | | **0.005** |  | | | **0.04** |  | | | 0.60 |
| Male | 30.0% | | 29.2% |  | 32.0% | | 27.2% |  | 31.6% | 27.6% |  | 25.9% | | 33.3% |  | 27.3% | | 31.9% |  | 27.8% | | 31.4% |  | 30.1% | | 29.1% |  |
| Female | 20.0% | | 20.8% |  | 17.8% | | 22.9% |  | 18.3% | 22.5% |  | 24.2% | | 16.6% |  | 22.7% | | 18.1% |  | 22.0% | | 18.7% |  | 19.9% | | 20.9% |  |
| **Histotypes** |  | | | 0.60 |  | | | **<0.001** |  | | **<0.001** |  | | | **<0.001** |  | | | **<0.001** |  | | | **<0.001** |  | | | 0.90 |
| ADC | 24.8% | | 25.8% |  | 19.4% | | 31.1% |  | 19.6% | 30.9% |  | 39.1% | | 11.5% |  | 36.8% | | 13.7% |  | 28.9% | | 21.7% |  | 25.4% | | 25.1% |  |
| SqCC | 25.2% | | 24.2% |  | 30.6% | | 18.9% |  | 30.4% | 19.1% |  | 10.9% | | 38.5% |  | 13.2% | | 36.3% |  | 21.1% | | 28.3% |  | 24.6% | | 24.9% |  |
| **T stage** |  | | | 0.47 |  | | | 0.24 |  | | **0.001** |  | | | 0.72 |  | | | 0.77 |  | | | 0.96 |  | | | 0.68 |
| T1 | 15.3% | | 13.5% |  | 15.5% | | 13.3% |  | 11.5% | 17.3% |  | 15.1% | | 13.8% |  | 14.8% | | 14.0% |  | 14.1% | | 14.7% |  | 15.2% | | 13.7% |  |
| T2 | 27.4% | | 30.0% |  | 27.0% | | 30.4% |  | 31.3% | 26.1% |  | 28.5% | | 28.9% |  | 28.3% | | 29.1% |  | 28.8% | | 28.6% |  | 27.7% | | 29.7% |  |
| T3 | 6.6% | | 5.8% |  | 6.7% | | 5.7% |  | 6.5% | 5.9% |  | 6.0% | | 6.3% |  | 6.3% | | 6.0% |  | 6.3% | | 6.0% |  | 6.3% | | 6.0% |  |
| T4 | 0.8% | | 0.6% |  | 0.8% | | 0.6% |  | 0.6% | 0.8% |  | 0.5% | | 0.9% |  | 0.5% | | 0.9% |  | 0.6% | | 0.8% |  | 0.8% | | 0.6% |  |
| **N stage** |  | | | 0.32 |  | | | 0.68 |  | | **0.03** |  | | | 0.41 |  | | | 0.06 |  | | | 0.59 |  | | | 0.85 |
| N0 | 34.2% | | 32.5% |  | 33.3% | | 33.5% |  | 31.7% | 35.0% |  | 33.0% | | 33.7% |  | 33.4% | | 33.4% |  | 32.4% | | 34.4% |  | 33.6% | | 33.2% |  |
| N1 | 10.9% | | 11.6% |  | 11.3% | | 11.1% |  | 13.1% | 9.4% |  | 11.1% | | 11.3% |  | 10.1% | | 12.3% |  | 11.5% | | 11.0% |  | 11.0% | | 11.5% |  |
| N2 | 4.7% | | 6.1% |  | 4.9% | | 5.9% |  | 5.3% | 5.5% |  | 6.1% | | 4.7% |  | 6.4% | | 4.4% |  | 5.8% | | 5.0% |  | 5.7% | | 5.1% |  |
| **Pathologic stage** |  | | | 0.29 |  | | | 0.53 |  | | 0.08 |  | | | 0.82 |  | | | 0.60 |  | | | 0.89 |  | | | 0.73 |
| I | 28.8% | | 26.3% |  | 27.4% | | 27.7% |  | 25.6% | 29.6% |  | 27.6% | | 27.5% |  | 26.8% | | 28.4% |  | 27.2% | | 27.9% |  | 27.0% | | 28.2% |  |
| II | 14.2% | | 15.7% |  | 15.6% | | 14.3% |  | 16.1% | 13.9% |  | 14.7% | | 15.3% |  | 15.3% | | 14.7% |  | 15.1% | | 14.9% |  | 15.3% | | 14.7% |  |
| IIIA | 6.9% | | 8.0% |  | 6.9% | | 8.0% |  | 8.1% | 6.8% |  | 7.8% | | 7.1% |  | 7.9% | | 7.0% |  | 7.7% | | 7.2% |  | 7.8% | | 7.1% |  |
| **Relapse** |  | | | 0.23 |  | | | **0.02** |  | | 0.23 |  | | | **0.009** |  | | | **<0.001** |  | | | 0.82 |  | | | 0.82 |
| No | 31.5% | | 34.1% |  | 35.5% | | 30.1% |  | 32.7% | 32.9% |  | 30.8% | | 34.8% |  | 29.2% | | 36.4% |  | 31.7% | | 33.9% |  | 33.1% | | 32.5% |  |
| Yes | 18.1% | | 16.3% |  | 15.5% | | 18.8% |  | 15.5% | 18.8% |  | 19.6% | | 14.8% |  | 20.6% | | 13.8% |  | 16.9% | | 17.4% |  | 17.1% | | 17.3% |  |
| **Radiotherapy** |  | | | 0.91 |  | | | 0.91 |  | | 0.24 |  | | | 0.14 |  | | | 0.67 |  | | | 0.34 |  | | | 0.46 |
| Yes | 5.8% | | 6.3% |  | 6.0% | | 6.0% |  | 5.3% | 6.7% |  | 6.9% | | 5.2% |  | 6.3% | | 5.8% |  | 6.5% | | 5.5% |  | 6.5% | | 5.5% |  |
| No | 43.1% | | 44.8% |  | 44.7% | | 43.3% |  | 44.2% | 43.8% |  | 42.9% | | 45.1% |  | 43.4% | | 44.6% |  | 42.8% | | 45.2% |  | 43.8% | | 44.2% |  |
| **Status** |  | |  | 0.95 |  | |  | 0.84 |  |  | 0.74 |  | |  | 0.74 |  | |  | 0.46 |  | |  | 0.20 |  | |  | 0.84 |
| Alive | 30.8% | | 31.0% |  | 31.1% | | 30.7% |  | 30.6% | 31.2% |  | 31.2% | | 30.6% |  | 30.3% | | 31.5% |  | 29.8% | | 32.0% |  | 31.1% | | 30.7% |  |
| Dead | 19.2% | | 19.0% |  | 18.9% | | 19.3% |  | 19.4% | 18.8% |  | 18.8% | | 19.4% |  | 19.7% | | 18.5% |  | 20.2% | | 18.0% |  | 18.9% | | 19.3% |  |

^a^Some cases has a missing data: Age (37); Gender (9); Histotypes (7); Pathologic stage (12); T stage (9); N stage (22); Relapse (148); Radiotherapy (107); Status (7).

**Abbreviations:** ADC, adenocarcinoma; SqCC, squamous cell carcinoma.
