# Supplementary material for: Modeling extracellular matrix through histo-molecular gradient in NSCLC for clinical decisions
Source: Front Oncol. 2022 Nov 14;12:1042766. doi: 10.3389/fonc.2022.1042766 (PMC9703002; doi:10.3389/fonc.2022.1042766)
Supplement: Supplementary Figure 1 — Negative controls of immunofluorescence for E-cadherin and β-catenin in the different histological subtypes of NSCLC. The stained nuclei are represented in blue (DAPI). Original magnification: 40X. LCC, large cell carcinoma; ADC, lung adenocarcinoma; SqCC: lung squamous cell carcinoma. [file DataSheet_1.zip › Table 3.DOCX]

**Supplementary Table 3.** Clinicopathologic characteristics and mean mRNA expression of heparan sulfate, chondroitin sulfate, and collagens types I, III, IV and V (TCGA database, N=939; Chi-square test, P<0.05)

| **Characteristics** | **Col I** | | **P** | **Col III** | | **P** | **Col IV** | | **P** | **Col V** | | **P** |
| --- | --- | --- | --- | --- | --- | --- | --- | --- | --- | --- | --- | --- |
|  | **Low** | **High** |  | **Low** | **High** |  | **Low** | **High** |  | **Low** | **High** |  |
| **Age (years)^a^** |  | | 0.79 |  | | 0.08 |  | | 0.62 |  | | 0.15 |
| ≤65 | 21.4% | 21.7% |  | 22.9% | 20.2% |  | 21.7% | 21.4% |  | 22.6% | 20.5% |  |
| >65 | 28.7% | 28.2% |  | 26.9% | 29.9% |  | 27.7% | 29.2% |  | 27.1% | 29.8% |  |
| **Gender ^a^** |  | | 0.51 |  | | 0.35 |  | | 0.95 |  | | 1.00 |
| Male | 29.0% | 30.2% |  | 28.8% | 30.4% |  | 29.5% | 29.8% |  | 29.7% | 29.6% |  |
| Female | 21.0% | 19.8% |  | 21.2% | 19.6% |  | 20.4% | 20.3% |  | 20.3% | 20.4% |  |
| **Histotypes ^a^** |  | | 0.36 |  | | **<0.001** |  | | 0.29 |  | | 0.15 |
| ADC | 26.1% | 24.5% |  | 28.5% | 22.0% |  | 24.4% | 26.2% |  | 26.5% | 23.5% |  |
| SqCC | 23.9% | 25.5% |  | 21.5% | 28.0% |  | 25.6% | 23.8% |  | 24.0% | 26.0% |  |
| **T stage ^a^** |  | | 0.40 |  | | 0.23 |  | | 0.14 |  | | 0.52 |
| T1 | 15.6% | 13.2% |  | 15.9% | 12.9% |  | 15.3% | 13.5% |  | 15.2% | 13.7% |  |
| T2 | 28.0% | 29.5% |  | 27.4% | 30.0% |  | 28.7% | 28.7% |  | 28.3% | 29.1% |  |
| T3 | 5.7% | 6.7% |  | 5.9% | 6.5% |  | 5.1% | 7.3% |  | 5.7% | 6.7% |  |
| T4 | 0.8% | 0.6% |  | 0.8% | 0.6% |  | 0.9% | 0.5% |  | 0.9% | 0.5% |  |
| **N stage ^a^** |  | | 0.15 |  | | 0.28 |  | | 0.39 |  | | 0.85 |
| N0 | 34.7% | 32.1% |  | 34.4% | 32.4% |  | 34.4% | 32.4% |  | 33.4% | 33.4% |  |
| N1 | 9.9% | 12.5% |  | 10.1% | 12.3% |  | 10.4% | 12.1% |  | 11.0% | 11.5% |  |
| N2 | 5.5% | 5.3% |  | 5.6% | 5.2% |  | 5.2% | 5.6% |  | 5.7% | 5.1% |  |
| **Pathologic stage ^a^** |  | | 0.14 |  | | 0.27 |  | | 0.25 |  | | 0.64 |
| I | 29.1% | 26.0% |  | 28.8% | 26.3% |  | 28.8% | 26.3% |  | 28.0% | 27.1% |  |
| II | 13.7% | 16.3% |  | 13.9% | 16.1% |  | 14.2% | 15.7% |  | 14.2% | 15.7% |  |
| IIIA | 7.1% | 7.8% |  | 7.2% | 7.7% |  | 6.8% | 8.1% |  | 7.6% | 7.3% |  |
| **Relapse ^a^** |  | | 0.60 |  | | 0.77 |  | | 0.18 |  | | 0.33 |
| No | 31.2% | 34.4% |  | 32.0% | 33.6% |  | 33.1% | 32.5% |  | 30.8% | 34.8% |  |
| Yes | 17.1% | 17.3% |  | 17.2% | 17.2% |  | 15.5% | 18.8% |  | 17.4% | 16.9% |  |
| **Radiotherapy ^a^** |  | | 0.17 |  | | 0.34 |  | | 0.39 |  | | 0.29 |
| Yes | 6.6% | 5.4% |  | 6.5% | 5.5% |  | 5.3% | 6.7% |  | 6.5% | 5.5% |  |
| No | 41.6% | 46.4% |  | 42.8% | 45.2% |  | 42.9% | 45.1% |  | 42.1% | 45.9% |  |
| **Status ^a^** |  |  | 0.20 |  |  | 0.54 |  |  | 0.31 |  |  | 0.74 |
| Alive | 32.0% | 29.8% |  | 31.4% | 30.4% |  | 31.8% | 30.0% |  | 31.2% | 30.6% |  |
| Dead | 18.0% | 20.2% |  | 18.6% | 19.6% |  | 18.2% | 20.0% |  | 18.8% | 19.4% |  |

^a^Some cases has a missing data: Age (37); Gender (9); Histotypes (7); Pathologic stage (12); T stage (9); N stage (22); Relapse (148); Radiotherapy (107); Status (7).

**Abbreviations:** ADC, adenocarcinoma; SqCC, squamous cell carcinoma; Col I, collagen type I; Col III, collagen type III, Col IV, collagen type IV; Col V, collagen type V.
