# Supplementary figures and images for: Modeling extracellular matrix through histo-molecular gradient in NSCLC for clinical decisions
Source: Front Oncol. 2022 Nov 14;12:1042766. doi: 10.3389/fonc.2022.1042766 (PMC9703002; doi:10.3389/fonc.2022.1042766)

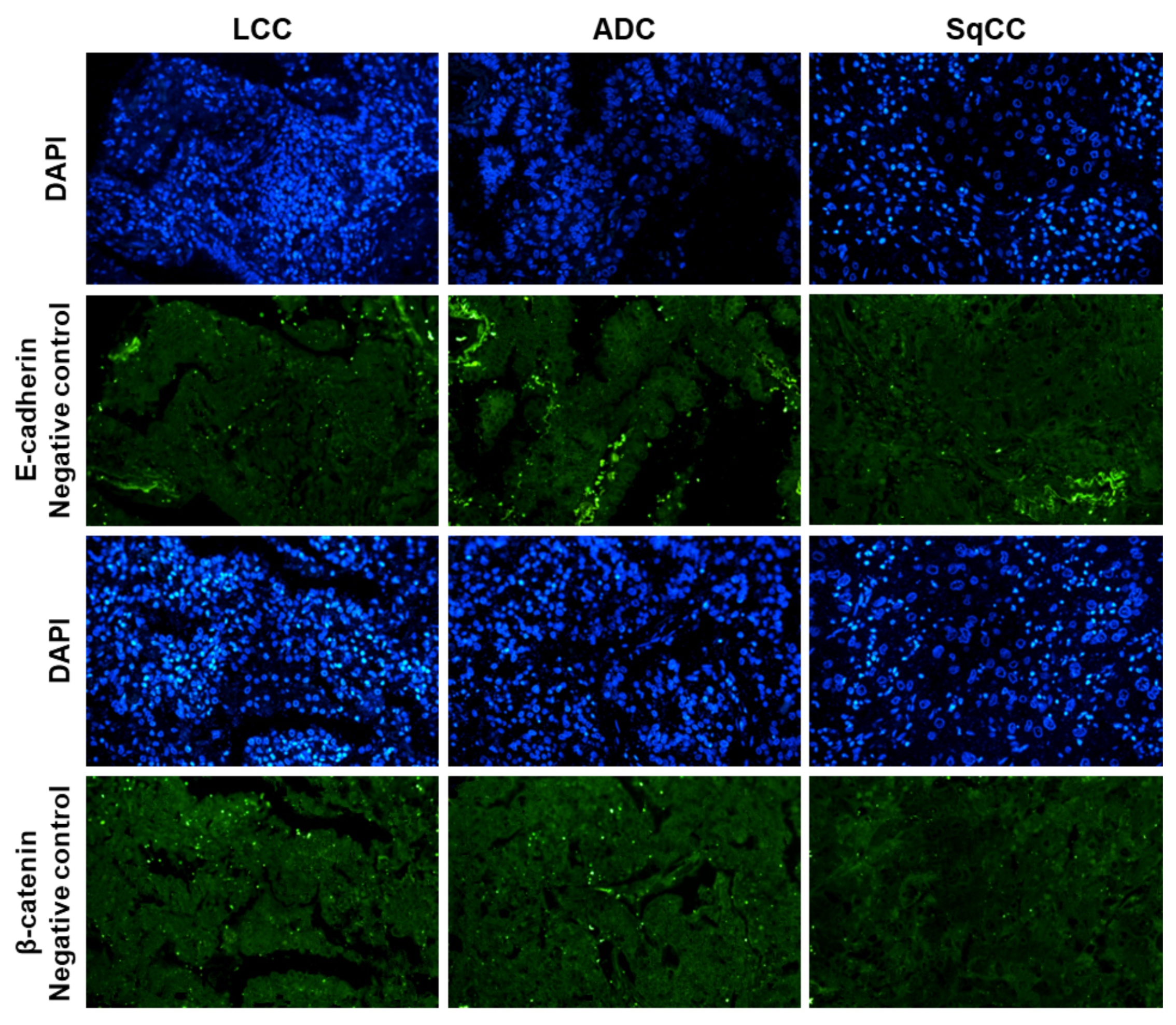

Supplement: Supplementary Figure 1 — Negative controls of immunofluorescence for E-cadherin and β-catenin in the different histological subtypes of NSCLC. The stained nuclei are represented in blue (DAPI). Original magnification: 40X. LCC, large cell carcinoma; ADC, lung adenocarcinoma; SqCC: lung squamous cell carcinoma. [file DataSheet_1.zip › Image 1.JPEG]

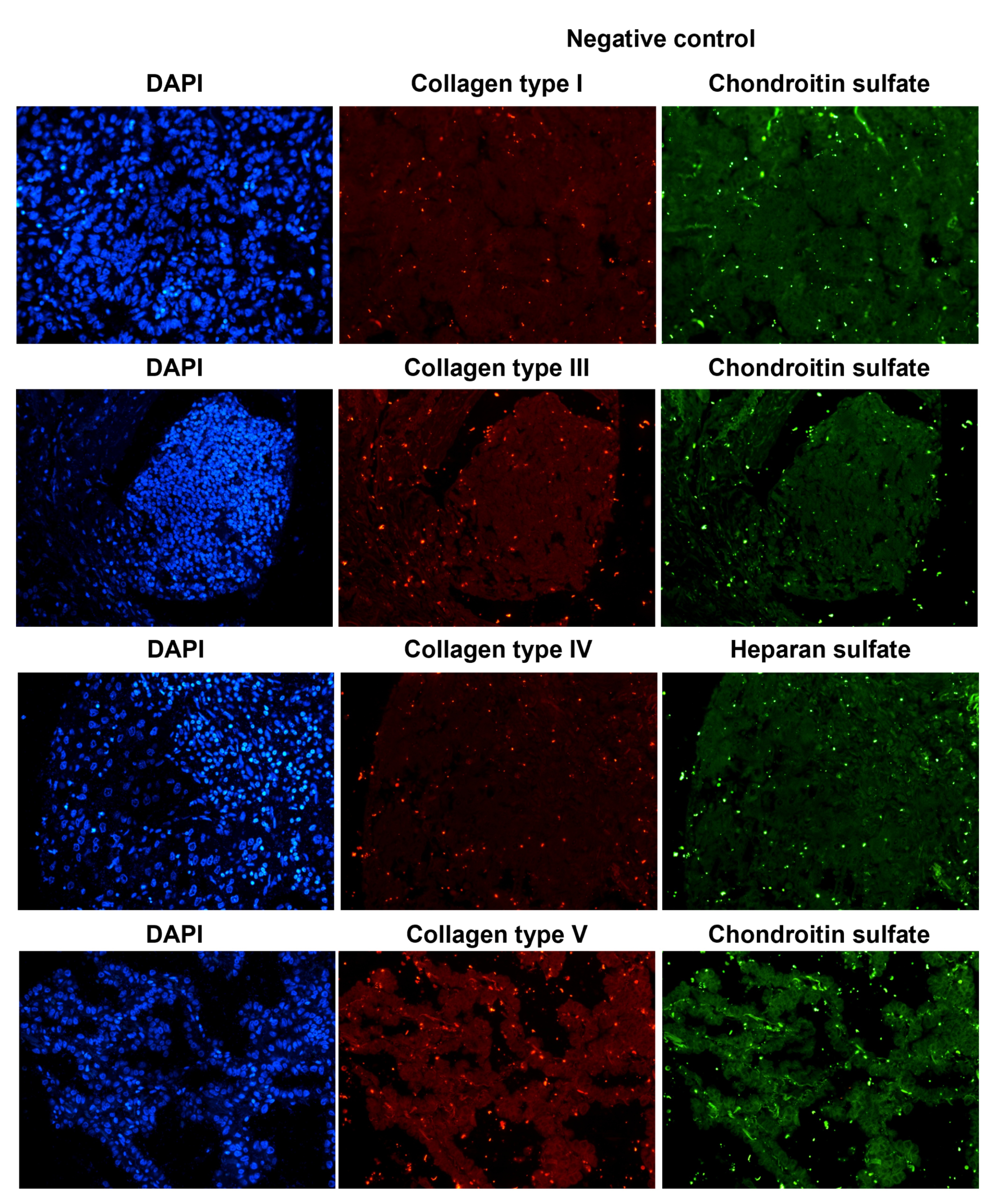

Supplement: Supplementary Figure 1 — Negative controls of immunofluorescence for E-cadherin and β-catenin in the different histological subtypes of NSCLC. The stained nuclei are represented in blue (DAPI). Original magnification: 40X. LCC, large cell carcinoma; ADC, lung adenocarcinoma; SqCC: lung squamous cell carcinoma. [file DataSheet_1.zip › Image 2.JPEG]

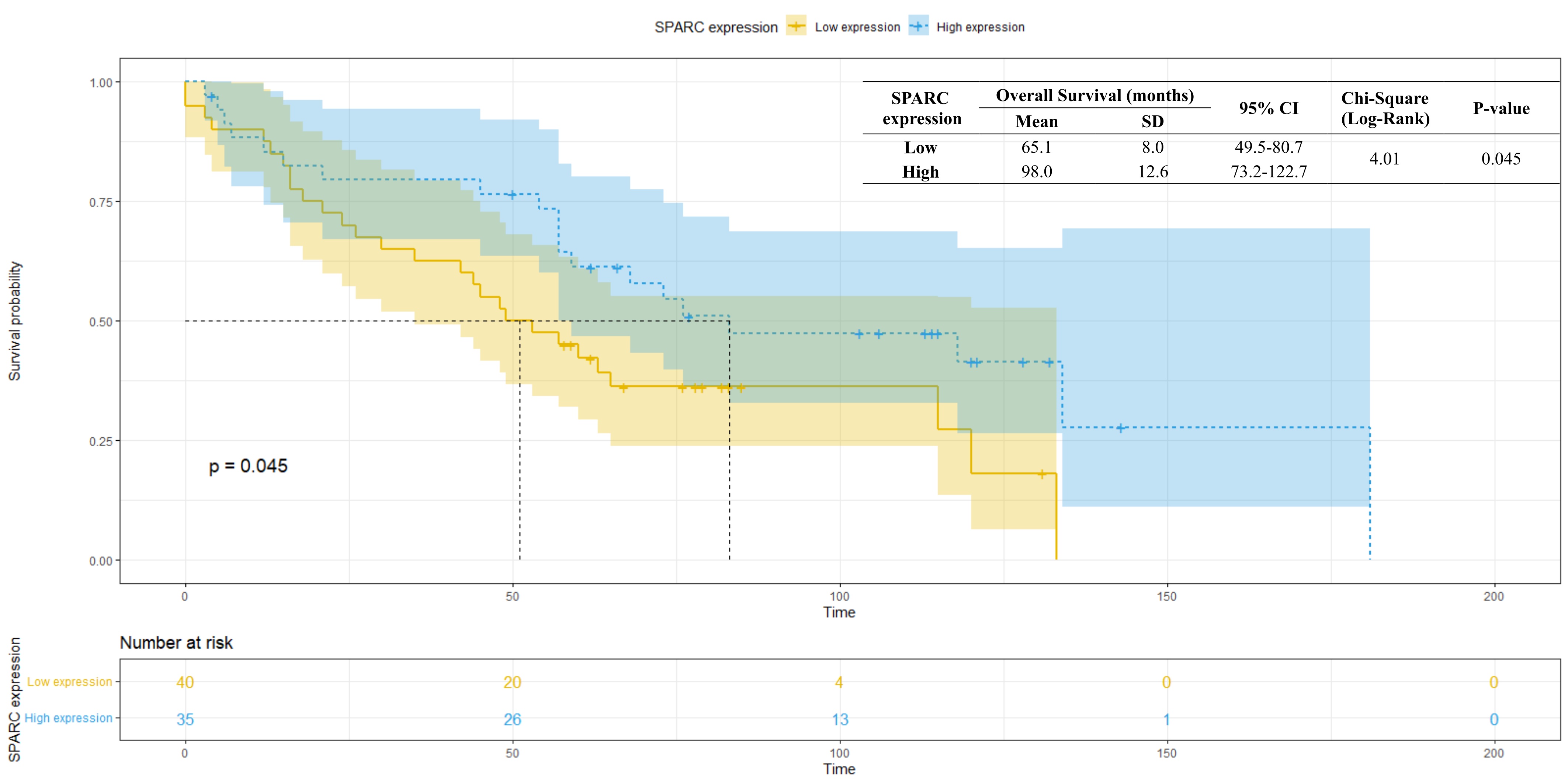

Supplement: Supplementary Figure 1 — Negative controls of immunofluorescence for E-cadherin and β-catenin in the different histological subtypes of NSCLC. The stained nuclei are represented in blue (DAPI). Original magnification: 40X. LCC, large cell carcinoma; ADC, lung adenocarcinoma; SqCC: lung squamous cell carcinoma. [file DataSheet_1.zip › Image 3.JPEG]

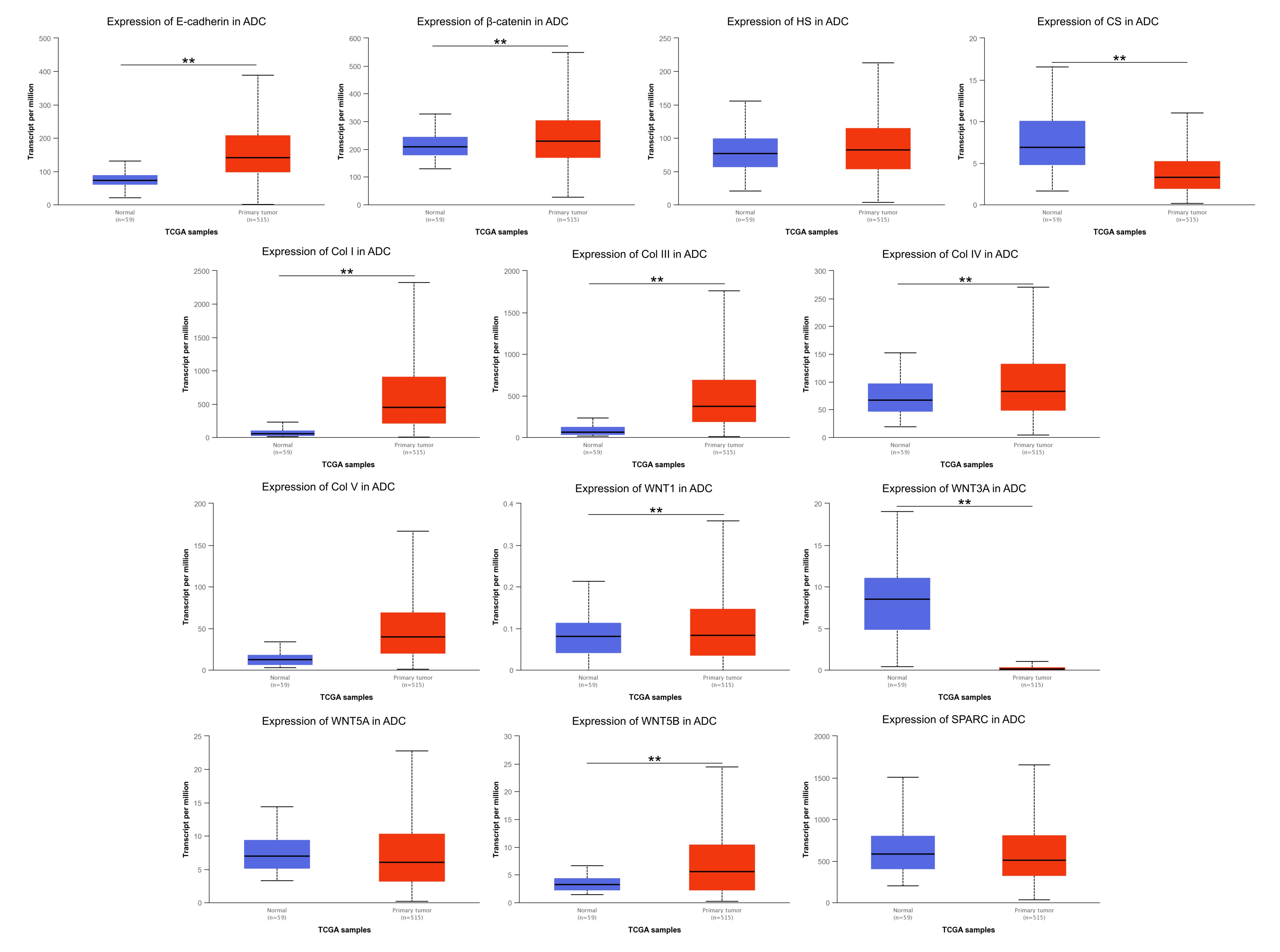

Supplement: Supplementary Figure 1 — Negative controls of immunofluorescence for E-cadherin and β-catenin in the different histological subtypes of NSCLC. The stained nuclei are represented in blue (DAPI). Original magnification: 40X. LCC, large cell carcinoma; ADC, lung adenocarcinoma; SqCC: lung squamous cell carcinoma. [file DataSheet_1.zip › Image 4.JPEG]

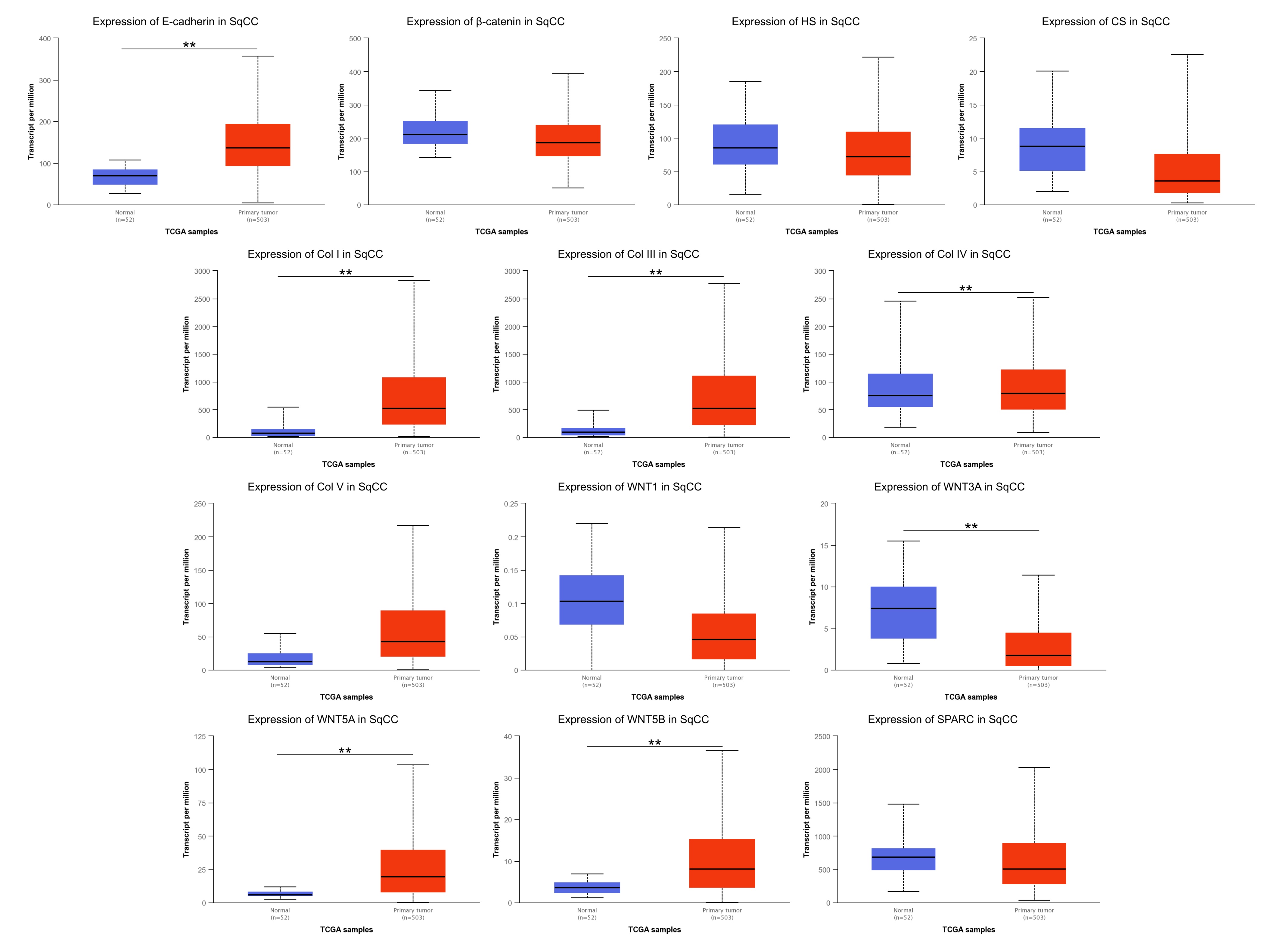

Supplement: Supplementary Figure 1 — Negative controls of immunofluorescence for E-cadherin and β-catenin in the different histological subtypes of NSCLC. The stained nuclei are represented in blue (DAPI). Original magnification: 40X. LCC, large cell carcinoma; ADC, lung adenocarcinoma; SqCC: lung squamous cell carcinoma. [file DataSheet_1.zip › Image 5.JPEG]

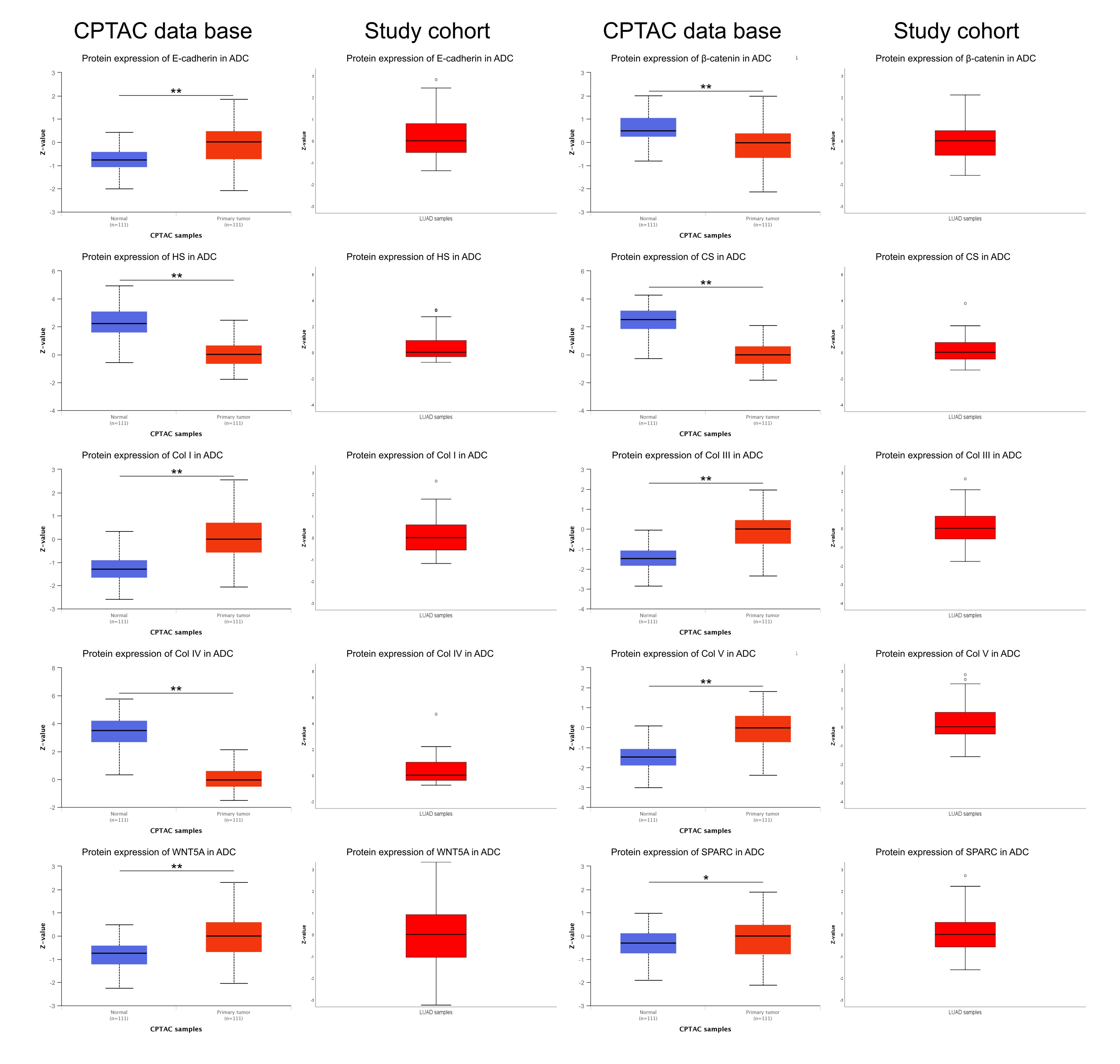

Supplement: Supplementary Figure 1 — Negative controls of immunofluorescence for E-cadherin and β-catenin in the different histological subtypes of NSCLC. The stained nuclei are represented in blue (DAPI). Original magnification: 40X. LCC, large cell carcinoma; ADC, lung adenocarcinoma; SqCC: lung squamous cell carcinoma. [file DataSheet_1.zip › Image 6.JPEG]

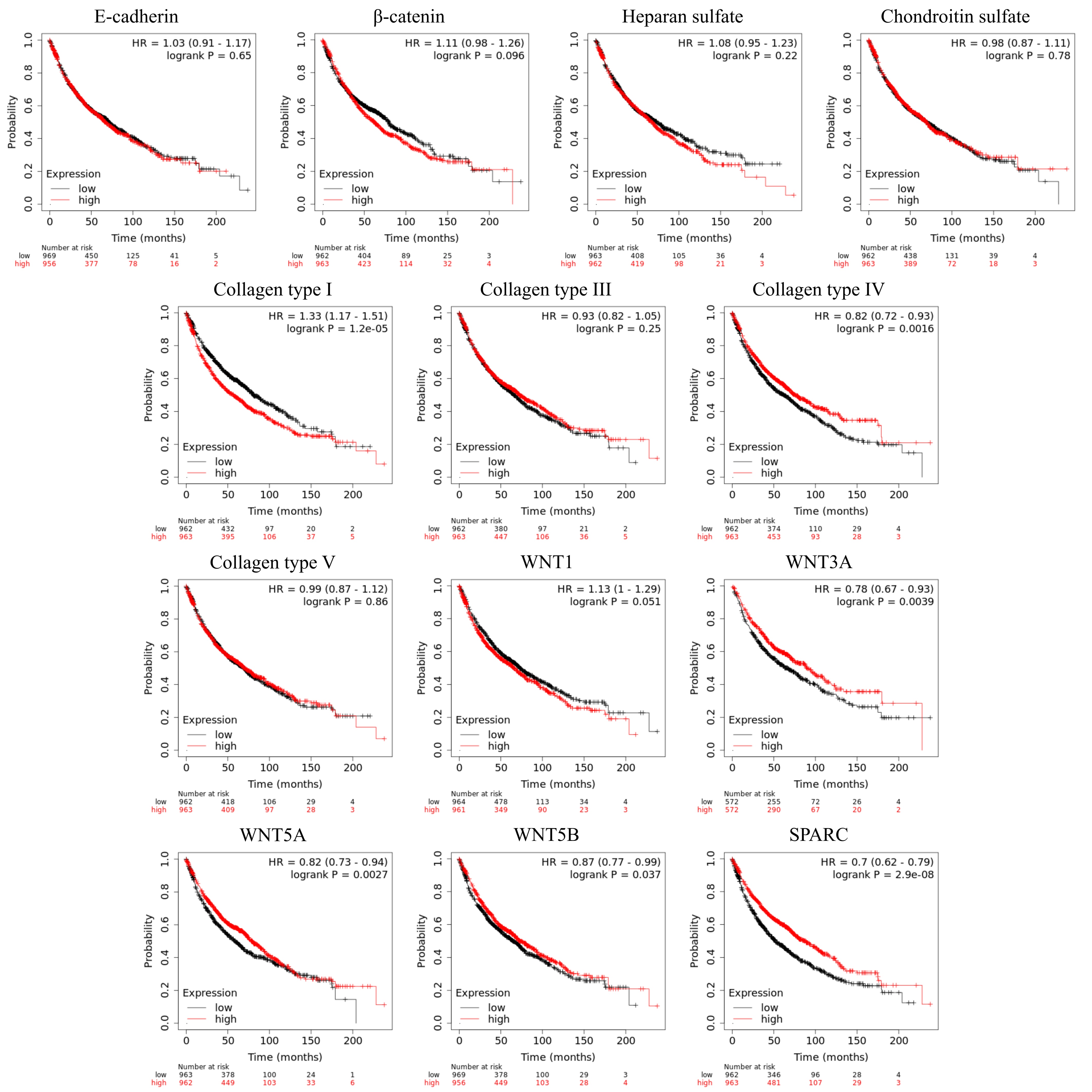

Supplement: Supplementary Figure 1 — Negative controls of immunofluorescence for E-cadherin and β-catenin in the different histological subtypes of NSCLC. The stained nuclei are represented in blue (DAPI). Original magnification: 40X. LCC, large cell carcinoma; ADC, lung adenocarcinoma; SqCC: lung squamous cell carcinoma. [file DataSheet_1.zip › Image 7.JPEG]

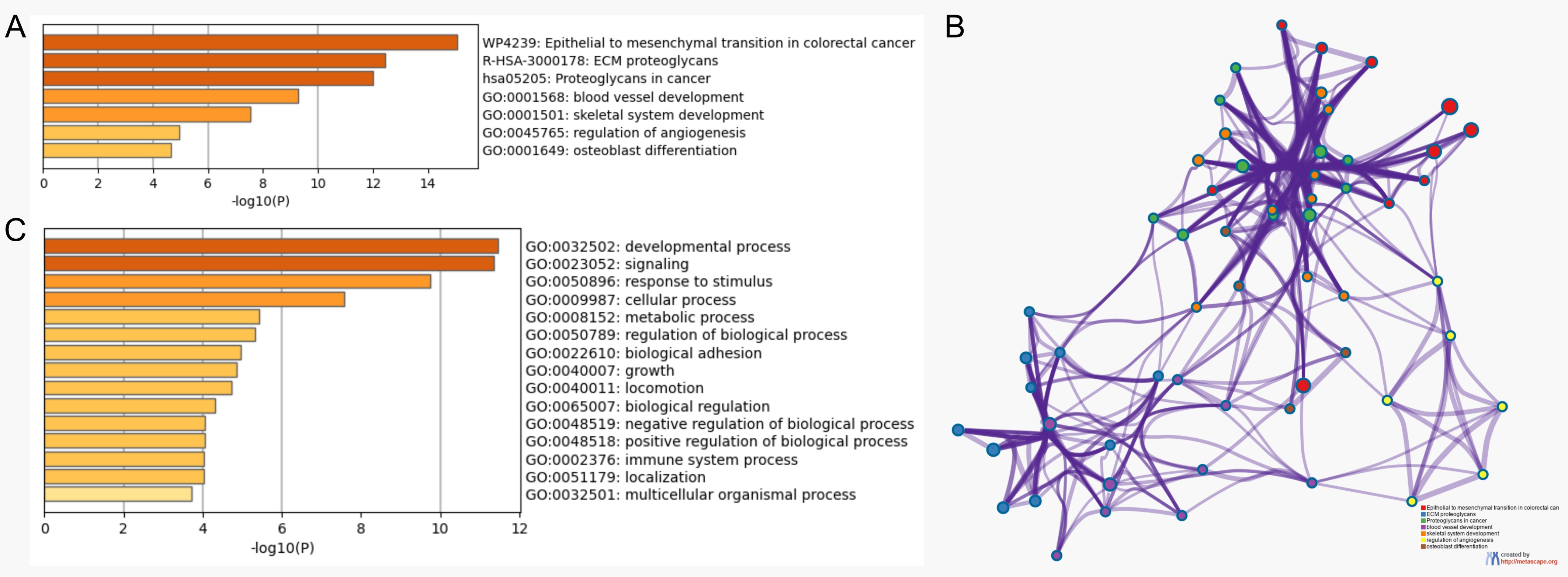

Supplement: Supplementary Figure 1 — Negative controls of immunofluorescence for E-cadherin and β-catenin in the different histological subtypes of NSCLC. The stained nuclei are represented in blue (DAPI). Original magnification: 40X. LCC, large cell carcinoma; ADC, lung adenocarcinoma; SqCC: lung squamous cell carcinoma. [file DataSheet_1.zip › Image 8.JPEG]
